# Bioassay-directed analysis-based identification of relevant pyrrolizidine alkaloids

**DOI:** 10.1007/s00204-022-03308-z

**Published:** 2022-05-24

**Authors:** Jochem Louisse, Patrick P. J. Mulder, Arjen Gerssen, Geert Stoopen, Deborah Rijkers, Milou G. M. van de Schans, Ad A. C. M. Peijnenburg

**Affiliations:** Wageningen Food Safety Research, Wageningen, The Netherlands

**Keywords:** Pyrrolizidine alkaloids, γH2AX assay, HepaRG cells, Bioassay, LC-MS/MS, LC-Orbitrap-MS

## Abstract

Pyrrolizidine alkaloids (PAs) are produced by various plant species and have been detected as contaminants in food and feed. Monitoring programmes should include PAs that are present in relevant matrices and that exhibit a high toxic potential. The aim of the present study was to use a bioassay-directed analysis approach to identify relevant PAs not yet included in monitoring programmes. To that end, extracts of *Heliotropium europaeum* and *H. popovii* were prepared and analysed with LC–MS/MS for the presence of 35 PAs included in monitoring programmes, as well as for genotoxic activity in the HepaRG/γH2AX assay. Europine, heliotrine and lasiocarpine were found to be the most abundant PAs. The extracts showed a higher γH2AX activity than related artificial mixtures of quantified known PAs, which might point to the presence of unknown toxic PAs. The *H. europaeum* extract was fractionated and γH2AX activities of individual fractions were determined. Fractions were further analysed applying LC–Orbitrap-MS analysis and Compound Discoverer software, identifying various candidate PAs responsible for the non-explained genotoxic activity. Altogether, the results obtained show that bioassay-directed analysis allows identification of candidate PAs that can be included in monitoring programmes.

**Supplementary Information:**

The online version contains supplementary material available at 10.1007/s00204-022-03308-z.

## Introduction

Pyrrolizidine alkaloids (PAs) are secondary metabolites produced as part of a defence strategy against insects by various plant species, particularly those belonging to the plant families Asteraceae (Compositae)*,* Boraginaceae and Fabaceae (Leguminosae) (Boppré 2011; Hartmann and Witte 1995; Liu et al. 2017). PAs have been reported to cause acute and chronic intoxication in livestock, wildlife and humans (Chen and Huo 2010; Chojkier 2003; Fu et al. 2004, Shimsoni et al. 2015). In developing countries, acute toxicity upon PA exposure has been reported in humans, causing severe intoxication including fatal incidents (Kakar et al. 2010; Robinson et al. 2014; Wiedenfeld 2011). Whereas risks of PA poisoning in humans are considered to be low in Europe, the discovery of substantial amounts of PAs in herbal infusions and teas increased concerns about possible health effects related to low chronic exposure (BfR 2013; Bodi et al. 2014; Chen et al. 2017; Mulder et al. 2015, 2018). Other frequently contaminated human food sources were found to be honey, milk and eggs (BfR 2013; Dübecke et al. 2011; EFSA 2011, 2017; Mulder et al. 2015, 2018). Of the several hundreds of PAs that have been identified, a subset of 17 PAs has initially been proposed by the EFSA CONTAM Panel to be monitored in food and feed. The Panel also recommended to include other PAs if possible, to better understand the occurrence of PAs in food and feed (EFSA 2017). Maximum levels of PAs in foodstuffs, including herbal infusions, teas, food supplements containing herbal ingredients including extracts, and others, have been recently set by the European Commission as described in the Commission Regulation (EU) 2020/2040 of 11 December 2020 amending Regulation (EC) No 1881/2006 as regards maximum levels of pyrrolizidine alkaloids in certain foodstuffs (EU 2020). These maximum levels have been set for 21 PAs and 14 PAs known to co-elute with one or more of these 21 PAs, being together the 35 PAs that have been selected by the European Commission.

**Fig. 1:**
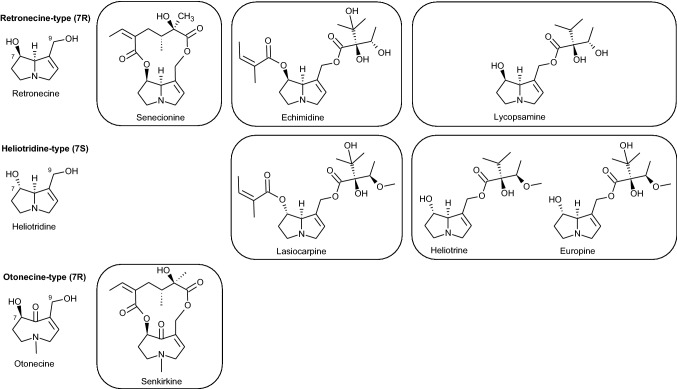
Chemical structures of some frequently occurring PAs. Unsaturated PAs can be divided in different categories according to their type of necine base (retronecine, heliotridine, otonecine) and their type of esterification (cyclic diesters, open diesters or monoesters). The necine base can be oxidised at the nitrogen atom, giving rise to PA N-oxides (not shown)

PAs consist of a pyrrolizidine nucleus (necine base: two fused, five-membered rings joined by a nitrogen atom) with side chains of various lengths and compositions attached to the C-7 and/or C-9 position (Fig. 1). Most PAs have a double bond between C-1 and C-2 of the necine base, which is required for their bioactivation and resulting toxicity. Unsaturated PAs are typically divided in different categories based on their type of necine base (retronecine (7R), heliotridine (7S), otonecine (7R) and their type of esterification (cyclic diesters, open diesters or monoesters) (Fig. 1). The necine base can be oxidised at the nitrogen atom, giving rise to PA *N*-oxides, which are the predominant PA forms in plants (Schrenk et al. 2020).

Health concerns of PAs are mainly related to the reported carcinogenicity of PAs in laboratory animals, which appears to be mediated via a genotoxic mode of action (Fu et al. 2004; see overview of reported animal studies in Chen et al. 2017). PAs have been shown to form DNA adducts, and cause DNA cross-linking, DNA double-strand breaks (DSBs), sister chromatid exchange, micronuclei, chromosomal aberrations, gene mutations and chromosome mutations in vivo and in vitro (Chen et al. 2010). When, for example, DSBs are inappropriately repaired, these can promote genetic instability and tumorigenesis (Khanna and Jackson 2001; Aparicio et al. 2014). To cause toxicity, PAs need to be bioactivated to highly reactive pyrrolic esters, which covalently bind with nucleophilic centres in glutathione, proteins or DNA (Ruan et al. 2014). Toxic potencies of different PAs have been reported to differ largely (Merz and Schrenk 2016), but available data indicate that in general, diester PAs are more toxic than monoester PAs. We recently determined the in vitro genotoxic potencies of 37 PAs in the γH2AX assay in human HepaRG liver cells. Of these 37 PAs, 26 PAs were positive in the assay with senecionine being the most potent and lycopsamine the least potent of the tested PAs showing γH2AX activity (Louisse et al. 2019).

From a health perspective, monitoring programmes should include those PAs that are present in relevant matrices (PA-containing plants that can contaminate food and feed) and that have a relatively high toxic potential. In theory, these may entail other PAs than the 35 PAs selected by the European Commission. The present study aims to assess whether a bioassay-directed analysis approach can be applied to identify such toxic PAs that would be of interest to be included in monitoring programmes. To that end, extracts of *Heliotropium europaeum* and *H. popovii* were prepared and analysed for the presence of known PAs and for γH2AX activity. In particular, *H. europaeum* is a widespread weed that is known to contaminate food and feed products (Mulder et al. 2018; Picron et al. 2021; Shimshoni et al. 2015). To assess whether unknown toxic PAs are present in the extracts, γH2AX activities of the extracts were compared with the γH2AX activities of related artificial mixtures of the quantified known PAs. In a next step, the extract of *H. europaeum* was fractionated and the activities of the fractions determined in the γH2AX assay. Finally, LC–Orbitrap-MS analysis and Compound Discoverer software were applied to the fractions to identify candidate PAs responsible for non-explained genotoxic activity.

## Materials and methods

### Chemicals

The following mono- and diester PAs were available for this study: echimidine, echimidine N-oxide, echinatine, echinatine N-oxide, europine, europine N-oxide, heliosupine, heliosupine N-oxide, heliotrine, heliotrine N-oxide, indicine, indicine N-oxide, intermedine, intermedine N-oxide, lasiocarpine, lasiocarpine N-oxide, lycopsamine, lycopsamine N-oxide, rinderine, and rinderine N-oxide. In addition, a set of macrocyclic PAs was included in the method (see Supplementary Table 1 for detailed supplier information and purity of all PA standards used for this study). Aflatoxin B1 (AFB1), which was used as a genotoxic reference compound in the γH2AX assay, was obtained from Sigma-Aldrich (Zwijndrecht, The Netherlands). All stock solutions of the compounds were prepared in 100% dimethyl sulfoxide (DMSO HybriMax, Sigma-Aldrich). Deuterated standards (7,9-dibutylretronecine-d_2_, 7,9-dibutylretronecine N-oxide-d_2_, 7,9-dibutylheliotridine-d_2_, 7,9-dibutylheliotridine N-oxide-d_2_) were custom synthesised by ChiroBlock (Bitterfeld-Wolfen, Germany). The standards are deuterated at the methylene position of the molecule with a d_2_-incorporation higher than 98%.

### HepaRG cell culture

The human hepatic cell line HepaRG was obtained from Biopredic International (Rennes, France) and cultured in growth medium consisting of William’s Medium E + GlutaMAX™ (ThermoFisher Scientific, Landsmeer, The Netherlands) supplemented with 10% Good Forte filtrated bovine serum (FBS; PAN™ Biotech, Aidenbach, Germany), 1% PS (100 U/ml penicillin, 100 µg/ml streptomycin; Capricorn Scientific, Ebsdorfergrund, Germany), 50 µM hydrocortisone hemisuccinate (sodium salt) (Sigma-Aldrich), and 5 µg/ml human insulin (PAN™ Biotech). Seeding, trypsinisation (using 0.05% Trypsin–EDTA (ThermoFisher Scientific)) and maintenance of the cells was performed according to the HepaRG instruction manual from Biopredic International. For toxicity studies (cell viability and genotoxicity studies), HepaRG cells were seeded in black-coated 96-well plates (Greiner Bio-One, Frickenhausen, Germany; 9000 cells per well in 100 µl). After 2 weeks on growth medium, cells were cultured for 2 days in growth medium supplemented with 0.85% DMSO to induce differentiation. Subsequently, cells were cultured for 12 days in growth medium supplemented with 1.7% DMSO (differentiation medium) for final differentiation. At this stage, cells were ready to be used for toxicity studies. Cells that were not immediately used were kept on differentiation medium for a maximum of three additional weeks. Cell cultures were maintained in an incubator (humidified atmosphere with 5% CO_2_ at 37 °C) and the medium was refreshed every 2–3 days during culturing. Prior to toxicity studies, differentiated HepaRG cells were incubated for 24 h in assay medium (growth medium containing 2% FBS) supplemented with 0.5% DMSO.

### Cell exposure

Cells were exposed for 24 h to individual PAs, artificial PA mixtures, and plant extracts. PAs and extracts were diluted from 200X-concentrated stock solutions in assay medium, providing a final DMSO concentration of 0.5%. In each experiment, a solvent control (0.5% DMSO) and a positive control (2.5 µM AFB1) were included. After exposure, effects of the PAs and extracts on cell viability and γH2AX induction were assessed. Each PA sample or plant extract was tested in two independent studies. In each study, each condition was tested in duplicate.

### Cell viability studies

The effect of the PAs and extracts on cell viability was determined using the WST-1 assay. This assay determines the conversion of the tetrazolium salt WST-1 (4-[3-(4-iodophenyl)-2-(4-nitrophenyl)-2H-5-tetrazolio]-1,3-benzene disulfonate) to formazan by metabolically active cells. After exposure for 24 h, the medium was removed and the cells were washed with Dulbecco’s Phosphate Buffered Saline (D-PBS; ThermoFisher Scientific). Next, WST-1 solution (Sigma-Aldrich) was added to the cell culture medium (1:10 dilution) and 100 µl was added to each well. After 1-h incubation in an incubator (humidified atmosphere with 5% CO_2_ at 37 °C), the plate was shaken at 1000 rpm for 1 min, and absorbance at 450 nm was measured (background absorbance at 630 nm was subtracted) using a microplate reader (Synergy™ HT BioTek, Winooski, VT, USA).

### γH2AX ICW assay

Genotoxic effects of PAs were determined using the γH2AX ICW assay, essentially as previously described (Audebert et al. 2010; Khoury et al. 2013; Louisse et al. 2019). After exposure for 24 h, the medium was removed and cells were washed with D-PBS. Then cells were fixed with 4% paraformaldehyde (ThermoFisher Scientific) in D-PBS. Subsequently, the cells were washed with D-PBS and incubated for 2 min with a 50 mM NH_4_Cl solution (Merck, Darmstadt, Germany). Subsequently, cells were washed with D-PBS, and permeabilised using 0.2% Triton™ X-100 (Sigma-Aldrich) in D-PBS, followed by a washing step with PST solution (0.2% Triton™ X-100 and 2% FBS in D-PBS). After permeabilisation, the cells were incubated for 1 h with MAXblock™ Blocking Medium (Active Motif, La Hulpe, Belgium) supplemented with phosphatase inhibitor PhosStop (Sigma-Aldrich) and bovine ribonuclease A (Sigma-Aldrich). This was followed by a 2-h incubation at room temperature with the primary antibody (Phospho-Histone H2A.X (Ser139) (20E3) Rabbit mAb, Cell Signaling Technology, Leiden, The Netherlands) in PST solution. Subsequently, cells were washed three times with PST solution, and incubated with an anti-goat antibody conjugated to an infrared fluorescent dye (Biotium, Fremont, CA, USA) and RedDot™ 2 (for DNA staining, Biotium) in PST solution. The RedDot2 signal is used as a measure for cell number, allowing normalisation of the γH2AX-response to cell number. After 1 h of incubation and subsequent three washes with PST solution, plates were scanned using an Odyssey Infrared Imaging System (LiCor ScienceTec, Les Ulis, France; Application Version 3.0). Raw data (integrated intensities, I.I (K counts)) were corrected for the background as described before (Khoury et al. 2013). Subsequently, the γH2AX/DNA fluorescence ratio of each well of the 96-well plate was determined (thereby normalising for the number of cells), and the fold change for each condition compared to the solvent control was determined by dividing the mean γH2AX/DNA fluorescence ratio by the mean γH2AX/DNA fluorescence ratio of the solvent control. Finally, the mean γH2AX induction and standard deviation of the biological duplicates were determined and these data were used for further assessment.

### Analysis of mixture effects

The mixture experiment was performed essentially as reported before for an in vitro study in which the effects of a mixture of three hepatotoxic pesticides were assessed in HepaRG cells (Lichtenstein et al. 2020). This latter study was executed within the EU project EuroMix (https://www.euromixproject.eu). The design of the mixture study was based on (1) estimation of the relative potency factors (RPFs) of the three most relevant PAs and (2) application of equipotent concentrations of these PAs in the mixture. Modelling of concentration–response data (γH2AX induction) and benchmark concentration (BMC)/RPF analysis of the individual PAs were performed using the PROAST benchmark dose modelling (BMD) webtool (PROASTweb version 67.0, RIVM, Bilthoven, Netherlands, https://proastweb.rivm.nl). In the PROAST webtool, concentration–response data are fitted to exponential and Hill models. The best fitted exponential and Hill model, i.e. having the lowest Akaike Information Criterion (AIC) value, are used for calculation of the RPF and the corresponding two-sided 90% confidence interval (CI) bounded by the RPFL (lower bound of the CI) and the RPFU (upper bound of the CI). RPF, RPFL, and RPFU, were determined for a benchmark response of 50% (BMR50) which corresponds to a 50% increase over the background level (γH2AX induction). The obtained RPF values were used as preliminary RPFs to calculate equipotent concentrations of the PAs for the mixture experiment. For the assessment of effects of the PA mixture, the concentration–response data for the mixture together with the data of the single PAs were analysed using the PROAST webtool. Thus, RPFs and their CIs were obtained for a first analysis without mixture data and compared with the RPFs and CIs derived from a second analysis including mixture data. In case RPFs from the first and second analysis are similar and the corresponding CIs overlap, dose addition can be assumed (Lichtenstein et al. 2020).

### Preparation of plant extracts

Common heliotrope (*Heliotropium europaeum*) was collected in the Luberon region, France in August 2014. *Heliotropium popovii* was collected in Afghanistan in 2008 during the toxic episode described by Kakar et al. (2010). Chamomile (*Matricaria recutita*) was collected in the vicinity of Wageningen, The Netherlands in 2017, which was used as a negative control (non-PA-containing plant). Materials were air-dried and subsequently milled and homogenised using a Peppink 200 AN Grinding machine (Veerman, Olst, The Netherlands). One-gram samples were transferred to 50 ml test tubes. 40 ml of a 2% formic acid (Merck) solution in water was added and the samples were extracted by rotary tumbling for 1 h. After centrifugation for 10 min at 3500 g, supernatants were collected and another portion of 15 ml 2% formic acid was added to the samples and the extraction was repeated. The two supernatants were combined, mixed and divided in two fractions of approximately 25 ml each. One fraction was chemically reduced by incubation with 10 mM Na_2_S_2_O_5_ (Sigma-Aldrich) for 1 h at room temperature on a rotary tumbler. The two fractions were subjected to SPE clean-up over StrataX 500 mg/6 ml cartridges (Phenomenex, Torrance, CA, USA). Cartridges were conditioned with 10 ml methanol (Biosolve, Valkenswaard, The Netherlands) followed by 10 ml water. After application of the extract, the cartridges were washed with 10 ml water. The SPE cartridges were dried by applying reduced pressure using a vacuum manifold for 5–10 min and the analytes were eluted with 10 ml methanol into polypropylene test tubes. The samples were evaporated under a gentle flow of nitrogen in a water bath kept at 50 °C (TurboVap, Zymark, Uppsala, Sweden) and the dry residues were reconstituted in DMSO at a ratio of approximately 250 µl of DMSO per gram dry weight. Reduced and non-reduced plant extracts were analysed by LC–MS/MS to determine the PA content. For LC–MS/MS analysis 0.5 µl DMSO extract was diluted with 100 µl methanol. Of this methanol extract, 10 µl was diluted with 490 µl water (final dilution of the DMSO extract: 10,000×).

### Analysis of PAs in plant extracts

Sample analysis was carried out using an LC–MS/MS system consisting of a Waters Acquity UPLC coupled to a Xevo TQ-S tandem mass spectrometer (Waters, Milford, MA, USA). The system was run in positive electrospray mode. Compounds were separated on a 150 × 2.1 mm 1.7 μm Acquity UPLC BEH C18 analytical column (Waters, Milford, MA, USA), kept at 50 °C and run at 0.4 ml min^−1^ with an acetonitrile/water gradient. Mobile phase A consisted of 10 mM (NH_4_)_2_CO_3_ (Honeywell Fluka, Landsmeer, The Netherlands) aqueous buffer at pH 9 and mobile phase B of pure acetonitrile (Biosolve). A gradient elution was performed as follows: 0.0 min 100% A/0% B, 0.1 min 95% A/5% B, 3.0 min 90% A/10% B, 7.0 min 76% A/24% B, 9.0 min 70% A/30% B, 12.0 min 30% A/70% B, 12.1–14.2 min 100% A/0% B. Of each sample extract, 2 μl was injected. See Supplementary Table 2 for the mass fragmentation settings used. Data were analysed using Targetlynx 4.2 software (Waters).

Standards in blank plant extract were used for quantification. To mimic a plant matrix background, alfalfa (*Medicago sativa*), a species which does not produce PAs, was used. 1 g of alfalfa was extracted in the same way as described above. Seven aliquots of 10 µl of the crude extract were transferred to HPLC vials and spiked with a mixture of the PAs standards and water was added to a final volume of 1 ml. The concentration range obtained (7 concentrations) was from 0 to 200 ng ml^−1^.

### Fractionation

Reduced *H. europaeum* extract was diluted 10 times with water and centrifuged 5 min, 14,000 rpm at room temperature. Fractions of the extract were prepared by injecting 100 µl of the clear supernatant on an Agilent 1200 series (G1314B) Diode Array HPLC system in several runs (measurement at 214 nm). Compounds were separated on an X-bridge prep C18 150 × 10 mm, 5 µm (Waters, Milford, MA, USA) semi-preparative column kept at 50 °C and run at 4 ml min^−1^ with an acetonitrile/water gradient. Mobile phase A consisted of water containing 10 mM (NH_4_)_2_CO_3_ (pH 9) and mobile phase B of pure acetonitrile. A gradient elution was performed as follows: 0.0 min 100% A/0% B, 0.5 min 95% A/5% B, 20 min 50% A/50% B, 21–23 min 20% A/80% B, 23–24 min 100% A/0% B, 24–30 min 100% A/0% B. Fractions were collected manually at the following time intervals: 1.5–3.5, 3.5–5, 5–6.5, 6.5–8, 8–10, 10–11.5, 11.5–13.5, 13.5–17, 17–19 and 19–23 min. In total, 850 µl was fractioned. Fractions of the different runs were pooled and concentrated under a flow of nitrogen in a water bath kept at 30 °C (Caliper TurboVap LV, MA, USA) until the percentage of acetonitrile was substantially reduced. The remaining fractions (30–60 ml) were further concentrated using SPE StrataX 500 mg/6 ml cartridges as described above. Before application of the extracts to the cartridges, the fractions were diluted with water to a total volume of 100 ml. The SPE cartridges were dried by applying reduced pressure using a vacuum manifold for 15 min and the analytes were eluted with 10 ml methanol into 10 ml test tubes. The samples were evaporated under a gentle flow of nitrogen in a water bath kept at 30 °C and the dry residues were reconstituted in 10 µl DMSO. The fractions were analysed by LC–MS/MS, using a calibration curve (7 concentrations, range 0–200 ng ml^−1^) of PA standards in water. Of the DMSO solutions, 5 µl was diluted with 100 µl methanol. Of this methanol solution, 20 µl was diluted with 980 µl water (final dilution of the DMSO extract: 1000×). Fractions 3, 5, 7 and 9 were additionally diluted 50-fold: 20 µl of the diluted DMSO extract was diluted with 980 µl water (final dilution of the DMSO extract: 50,000x). The fractions were also tested in the yH2AX assay. For that, 1.75 µl DMSO samples (and 3× and 9× dilutions in DMSO) were added to 348 µl culture medium (final DMSO concentration 0.5%). Moreover, the 10 fractions of *H. europaeum* were analysed for the presence of so far unknown PAs using LC–Orbitrap-MS analysis (see below). For LC–Orbitrap-MS analysis, the 1000-fold diluted DMSO extracts were used.

### Analysis of PA necine bases in fractions

For the analysis of the HPLC fractions, 2 µl of the 1000-fold diluted DMSO extract was diluted with 1 ml water. The solutions were spiked with 1 nmol ml^−1^ IS mixture consisting of four deuterated butyl diester analogues of retronecine and heliotridine and their corresponding N-oxides. The samples were subjected to alkaline hydrolysis by addition of 1 ml 0.6 N sodium hydroxide and heating in a water bath for 3 h at 90 °C. After cooling to room temperature, the pH of the solutions was adjusted to pH 2 by addition of 5 ml 1% formic acid solution. The samples were concentrated by SPE using StrataX SCX 200 mg/6 ml cartridges (Phenomenex). The cartridges were conditioned with 6 ml methanol followed by 6 ml 0.4% formic acid. After application of the extract the cartridges were washed with 6 ml 0.4% formic acid, followed by 6 ml methanol. The SPE cartridges were dried under vacuum and analytes were eluted with 6 ml 1.25% ammonia solution in methanol. The solvent was evaporated as described above and the residues were dissolved in 500 µl water.

Sample analysis was carried out using the same LC–MS/MS system as described above. The system was run in positive electrospray mode. Compounds were separated on a 150 × 2.1 mm 2.1 μm Astec® Chirobiotic^®^ R analytical column (Sigma-Aldrich, Zwijndrecht, The Netherlands), kept at 35 °C and run at 0.4 ml min^−1^ with a 4 mM NH_4_OAc buffer (Sigma-Aldrich) at pH 4.5. Of each sample extract, 5 μl was injected. See Supplementary Table 3 for the mass fragmentation settings used.

Calibration standards were prepared using heliotrine, heliotrine N-oxide, lycopsamine and lycopsamine N-oxide as the unlabeled reference compounds. Mixed solutions of these compounds (9 concentrations, range: 0–10 nmol ml^−1^) were combined with the deuterated internal standards (1 nmol ml^−1^) and then subjected to the same hydrolysis and concentration procedure as described above.

### Bioassay-directed identification

Collected *H. europaeum* fractions were analysed for the presence of possible new PAs using a combination of the γH2AX assay (applied to HepaRG cells) and LC–Orbitrap-MS. The LC system used was an Ultimate 3000 RS UHPLC system consisting of a quaternary pump, an autosampler and a column oven (Thermo Scientific, San Jose, CA, USA). Compounds were separated on a 150 × 2.1 mm 1.7 μm Acquity UPLC BEH C18 analytical column (Waters), kept at 50 °C and run at 0.4 ml min^−1^ with an acetonitrile/water gradient. Mobile phase A consisted of water containing 10 mM (NH_4_)_2_CO_3_ (pH 9) and mobile phase B of pure acetonitrile. A gradient elution was performed as follows: 0.0 min 100% A/0% B, 0.1 min 91% A/9% B, 3.0 min 86% A/14% B, 7.0 min 74% A/26% B, 9.0 min 58% A/42% B, 14.0 min 90% A/10% B, 14.1–16.2 min 100% A/0% B. The injection volume of the 1000-fold diluted DMSO extracts was 5 µl. The first minute of the separation was discarded to the waste, to avoid contamination of the MS interface. The eluent of the LC was interfaced with a HESI-II electrospray source coupled to a Q-Exactive Orbitrap mass spectrometer (Thermo Scientific, San Jose, CA, USA). The HESI-II source operated in positive ionisation mode, the capillary temperature was set at 250 °C with a spray voltage of 3.5 kV. A full scan with wide-isolation variable data independent acquisition (FS-vDIA) MS/MS method was applied. A total of seven scan events were applied. One full scan data were recorded with a *m/z* range of 200–550 with a resolution setting of 70,000, the automatic gain control (AGC) set at 3e6 and the maximum injection time (IT) set at 100 ms. Six vDIA scan events were set all with a resolution of 17,500, an AGC of 1e6 and an IT of 75 ms. In the vDIA scan events the precursor ion ranges were *m/z* 220–340 with a normalised collision energy (NCE) of 45, *m/z* 220–340 (NCE 30), m/z 330–430 (NCE 40), *m/z* 330–430 (NCE 25), *m/z* 420–550 (NCE 35) and *m/z* 420–550 (NCE 20). Data processing was performed using Thermo Scientific Compound Discoverer 3.1. In brief, various data processing nodes were applied in Compound Discoverer (Supplementary Fig. 1). Spectra were retrieved (“Select Spectra” node) from the raw data files for further processing. The software selected spectra should at least have a total intensity of 1e5 and a signal to noise of 3. After spectra selection, the node “Detect compounds” elucidated the potential compounds present. The following criteria were applied: solely the protonated ions should be detected as no other potential adducts were expected, and the molecular weight extracted should at least have a mw 133.0528 (corresponding to an elemental composition of C_8_H_7_NO) and should not exceed a molecular weight of 640.3333 (elemental composition of C_32_H_50_NO_12_). The detected peaks should at least have a width of 0.2 min and contain 5 scans. Detected compounds were merged by the molecular weight and retention time across all processed data files by the “Group compound” node. If there were missing peaks (data points) in the detected compounds this was repaired using the node “Filling gaps”. The background compounds were detected by analysis of blank samples and the area of the detected compounds in the samples of interest should be tenfold higher than the area in the blank. The detected compounds were further searched in the nodes “Search mzCloud”, “Search ChemSpider”, “Search Mass Lists” and “Predict Compositions”. For the “search mzCloud” node, data of both the MS and DIA spectra were submitted and searched against existing spectra in the online mzCloud database. For “Search ChemSpider” the generated formulas or masses were submitted to selected sub libraries in ChemSpider (ACToR: Aggregated Computational Toxicology Resource; EPA DSSTox; EPA Toxcast; FDA UNII—NLM; FooDB; Toxin, Toxin-Target Database). “Search mass lists” is an off line search against an in-house created mass list, which contains a large set of open chain mono- and diester PAs (Supplementary Table 4). This list is based on theoretical combinations of necic acids and necine bases as reported for Boraginaceae species (El-Shazly and Wink 2014). With the “Elemental compositions” node predictions were based on a minimal elemental composition of C_8_H_7_NO and maximum elemental composition of C_32_H_50_NO_12_ and a ring and double bound equivalent (RBDE) between 2 and 12. For all four types of searches, the mass tolerance was set at 5 ppm. For the overview, the “assign compound annotation” node annotated compounds were based on 1st the Mass List search, 2nd mzCloud, 3rd predicted compositions and 4th ChemSpider search results. Results were exported to Microsoft Excel for expert judgement and further sorting. To have more confidence in the assigned compounds a second MS analysis was performed to obtain precursor specific fragmentation data. The applied chromatography and the source settings were as described above. However, the Q-Exactive Orbitrap mass spectrometer operated in data-dependent MS^2^ (ddMS^2^) mode. The in-house created mass list (Supplementary Table 4) was used as inclusion list. In ddMS^2^, when a *m/z* corresponding to the protonated ion of the elemental composition from the inclusion list is detected by the mass spectrometer, a MS^2^ fragmentation spectrum is collected. The specific protonated mass was isolated with an isolation width of 1 *m/z* and fragmented with a stepped NCE of 25 and 45. The ddMS^2^ spectra were recorded from *m*/*z* 50–1000 at a resolution of 17,500. The ddMS^2^ spectra obtained were examined with the Qual Browser window of Xcalibur 4.2 software (Thermo Scientific, San Jose, CA, USA).

## Results

### Analysis of PAs in plant extracts

Extracts of *H. europaeum* and *H. popovii* were prepared with a solution of 2% formic acid in water and part of the extracts was treated with sodium metabisulfite to reduce N-oxides to the free bases followed by SPE clean up. Subsequently, both the reduced and non-reduced extracts were analysed by LC–MS/MS to determine the level of the 35 PAs identified by the European Commission as being relevant for monitoring in food, including the 17 PAs proposed by the European Food Safety Authority. This analysis showed that both extracts contained 12 of the 35 PAs (Table 1) and that the predominant PAs in the non-reduced extracts were the N-oxides of europine, heliotrine and lasiocarpine and that a large part of the N-oxides, but not all, was converted to the free bases after reduction of the extracts (Table 1). The heliotridine-type (7S) monoesters europine and heliotrine and open-diester lasiocarpine were the most abundant PAs in the reduced extracts.

**Table 1:** PA content (mg/l) of reduced and non-reduced extracts in DMSO of *H. europaeum*, *H. popovii* and chamomile (*M. recutita*), which was used as negative control

	Total 35 PAs	Major 6 PAs^a^	Minor 29 PAs^b^	Echinatine	Echinatine N-oxide	Rinderine	Rinderine N-oxide	Heliotrine	Heliotrine N-oxide	Europine	Europine N-oxide	Heliosupine	Heliosupine N-oxide	Lasiocarpine	Lasiocarpine N-oxide
*H. europaeum* (non-reduced)	40,680	40,358	322	8	32	12	202	359	16,980	884	19,703	8	60	23	2409
*H. europaeum* (reduced)	19,585	19,407	179	14	14	57	68	2820	2498	6709	6987	13	13	174	220
*H. popovii* (non-reduced)	67,872	67,351	521	9	234	7	261	962	39,605	421	26,289	5	4	5.3	71
*H. popovii* (reduced)	38,115	37,759	355	136	40	122	46	13,043	2797	16,629	5271	5	7	10	9
*M. recutita*^c^	ND	ND	ND	ND	ND	ND	ND	ND	ND	ND	ND	ND	ND	ND	ND

*ND* not detectable

^a^Major 6 PAs: heliotrine, heliotrine N-oxide, europine, europine N-oxide, lasiocarpine, lasiocarpine N-oxide

^b^Minor PAs detected: echinatine, echinatine N-oxide, rinderine, rinderine N-oxide, heliosupine, heliosupine N-oxide

^c^Reduced and non-reduced extracts

### Effect analysis of plant extracts and an artificial mixture of PAs in the γH2AX assay

In our previous work, the HepaRG/γH2AX assay was demonstrated to be a useful tool to determine the genotoxic potencies of PAs (Louisse et al. 2019). After BMD modelling of the concentration–response data of a series of 37 PAs, the BMC_50_ values of the different PAs were determined and relative potency factors (RPFs) were calculated by dividing the BMC_50_ of the index PA riddelliine with the BMC_50_ of each PA. On the basis of the in vitro RPFs, which ranged from ≤ 0.01 to 1.2, four different potency classes were distinguished (group 1: RPF 0.3–1.2, group 2: RPF 0.1–0.3, group 3: RPF 0.01–0.1, group 4: RPF ≤ 0.01). Regarding the most abundant PAs in the *H. europaeum* and *H. popovii* extracts (Table 1), lasiocarpine is relatively potent (group 1) and europine and heliotrine have a lower potency (group 3). All PA N-oxides tested were negative in the HepaRG/γH2AX assay (Louisse et al. 2019).

**Fig. 2:**
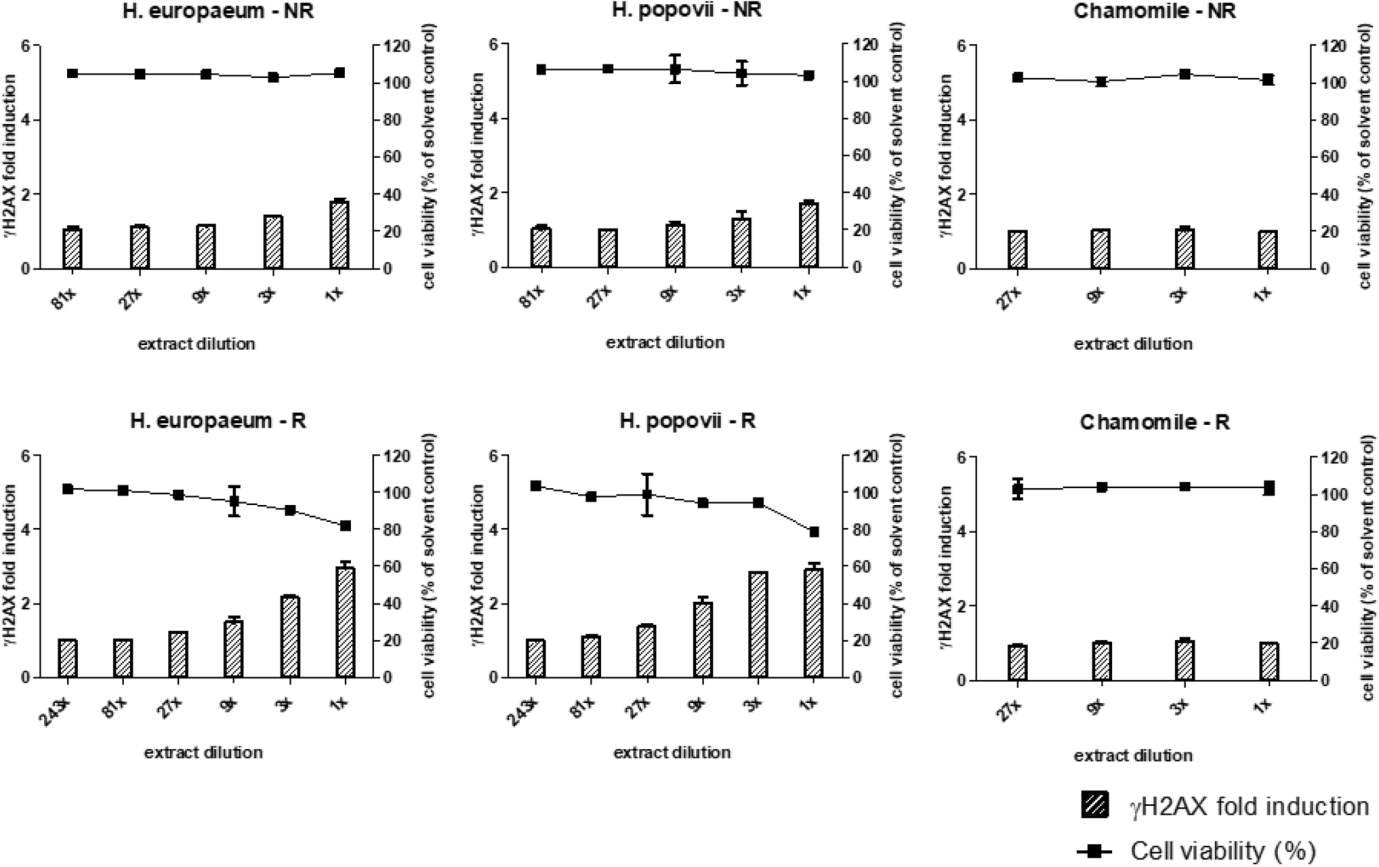
The effect of increasing concentrations of reduced (R) and non-reduced (NR) extracts of *H. europaeum*, *H. popovii* and chamomile (*M. recutita*) on viability of HepaRG cells (squares, right *Y*-axes) and γH2AX induction (bars, left *Y*-axes). For each condition, mean values (± SD) from two independent experiments are presented

In the present study, the HepaRG/γH2AX assay was applied to test reduced and non-reduced extracts of *H. europaeum*, *H. popovii* and *M. recutita.* In parallel, the effect of the extracts on cell viability was determined. A small reduction of cell viability was observed only for the highest concentrations of the reduced extracts of *H. europaeum* and *H. popovii* (Fig. 2). Both reduced and non-reduced *Heliotropium* extracts showed a concentration-dependent increase of γH2AX (at non-cytotoxic concentrations) with the reduced extracts showing the largest effect. In contrast, reduced and non-reduced extracts of *M. recutita* did not result in induction of γH2AX. These outcomes are in line with the data on PA-levels in the samples (Table 1), showing that the extracts with the highest level of free base PAs, i.e. the reduced extracts, were most potent in the γH2AX assay, and the PA-free *M. recutita* extracts did not induce γH2AX. These results and previously obtained γH2AX data for individual PAs including their N-oxides (Louisse et al. 2019) indicate that the γH2AX signal observed for the *Heliotropium* extracts is most likely due to the presence of free base PAs.

**Fig. 3:**
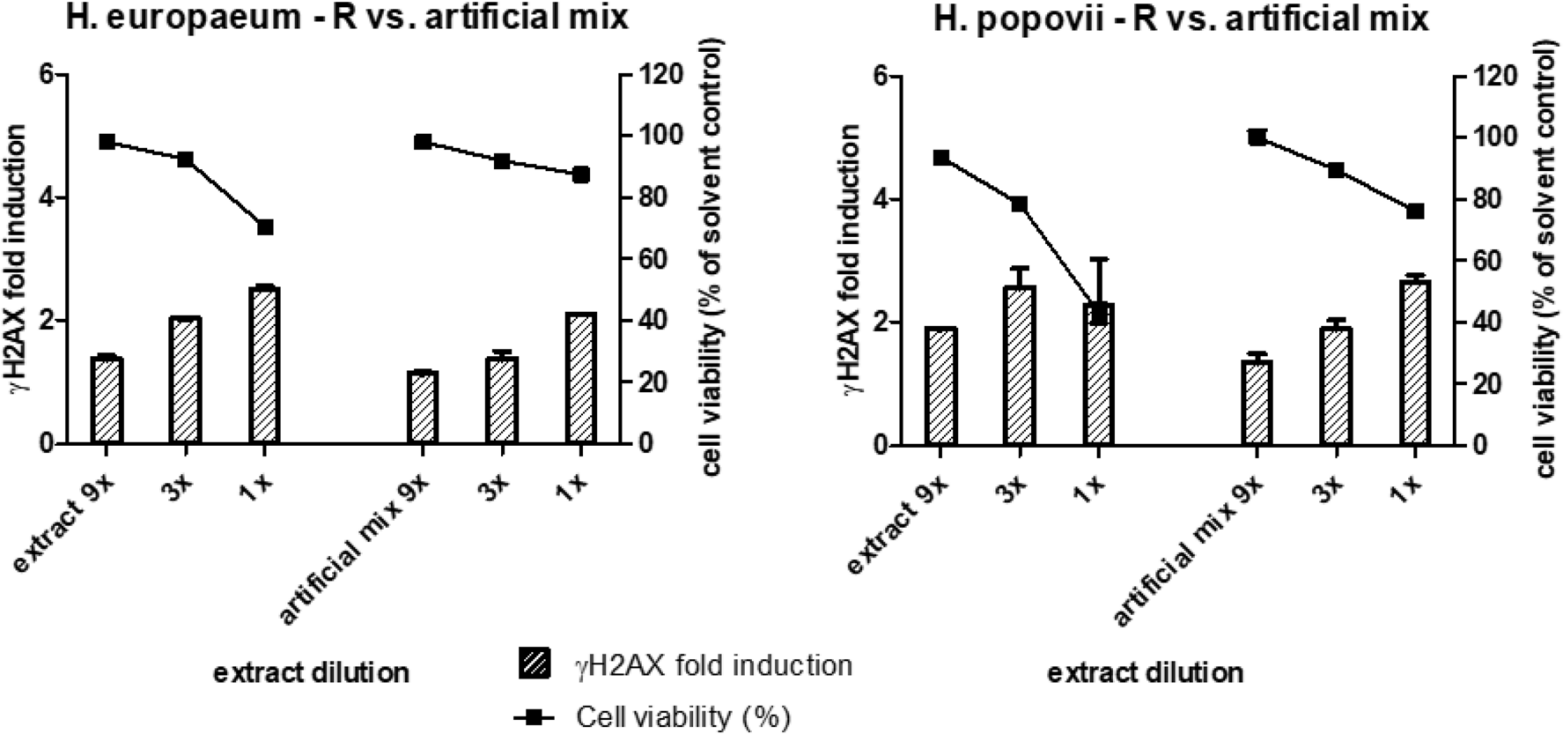
Comparison of the effects of reduced extracts of *H. europaeum* and *H. popovii* and artificial PA mixtures on cell viability and γH2AX induction. HepaRG cells were exposed to different dilutions (up to 9× diluted) of plant extracts and artificial mixtures in DMSO and analysed for their effects on cell viability (squares, right *Y*-axes) and γH2AX induction (bars, left *Y*-axes). For each condition, mean values (± SD) from two independent experiments are presented

In a follow-up experiment, reduced extracts of *H. europaeum* and *H. popovii* were tested in the HepaRG/γH2AX assay and compared with the effects of defined related artificial PA mixtures. This experiment was designed to be able to compare the γH2AX signals elicited by the extracts and the artificial mixtures and to conclude on the possible presence of so far unknown (potent) PAs. The artificial mixtures were prepared in DMSO and consisted of the three most abundant *Heliotropium* PAs, i.e. europine, heliotrine and lasiocarpine, at concentrations equal to those found in the DMSO-solubilised reduced plant extracts. The concentrations of europine, heliotrine and lasiocarpine were 6.71, 2.82, and 0.18 mg/ml DMSO, respectively, for the reduced *H. europaeum* extract and 16.63, 13.04, and 0.01 mg/ml DMSO, respectively, for the reduced extract of *H. popovii.* Since the levels of other PAs measured in the plant extracts were very low (Table 1), these were not included in the artificial mixtures, as these low levels are not expected to contribute to the γH2AX signal. As shown in Fig. 3, at identical dilutions, the γH2AX signal of the plant extracts was higher than the signal of the artificial mixtures. This may indicate that other PAs (other than the PAs measured for) have contributed to the relatively high γH2AX signal in the extracts.

### Analysis of mixture effects of PAs

For the identification of the compounds (particularly PAs) that are responsible for the larger induction of γH2AX by the *Heliotropium* extracts as compared to the artificial PA mixtures, it was decided to use a bioassay-directed analysis approach. Essential for using such an identification strategy is to assure that mixtures of PAs follow the concept of concentration addition (dose addition), i.e. that the combined effect is determined by the sum of the concentrations of the PAs in the mixture, corrected for differences in relative toxicity potency. Although dose addition is expected to apply to substances acting via a similar mode of action, to the best of our knowledge this has, so far, not been examined for PAs. Therefore, a mixture experiment was performed to investigate, using γH2AX induction as read-out, whether dose addition occurs for PAs.

Prior to this mixture study, first preliminary RPFs for each of the individual PAs were determined on the basis of the concentration–response data generated in the HepaRG/γH2AX assay. The concentration–response data and outcome of the BMD modelling are shown in Supplementary Fig. 2 and Supplementary Fig. 3, respectively.

**Fig. 4:**
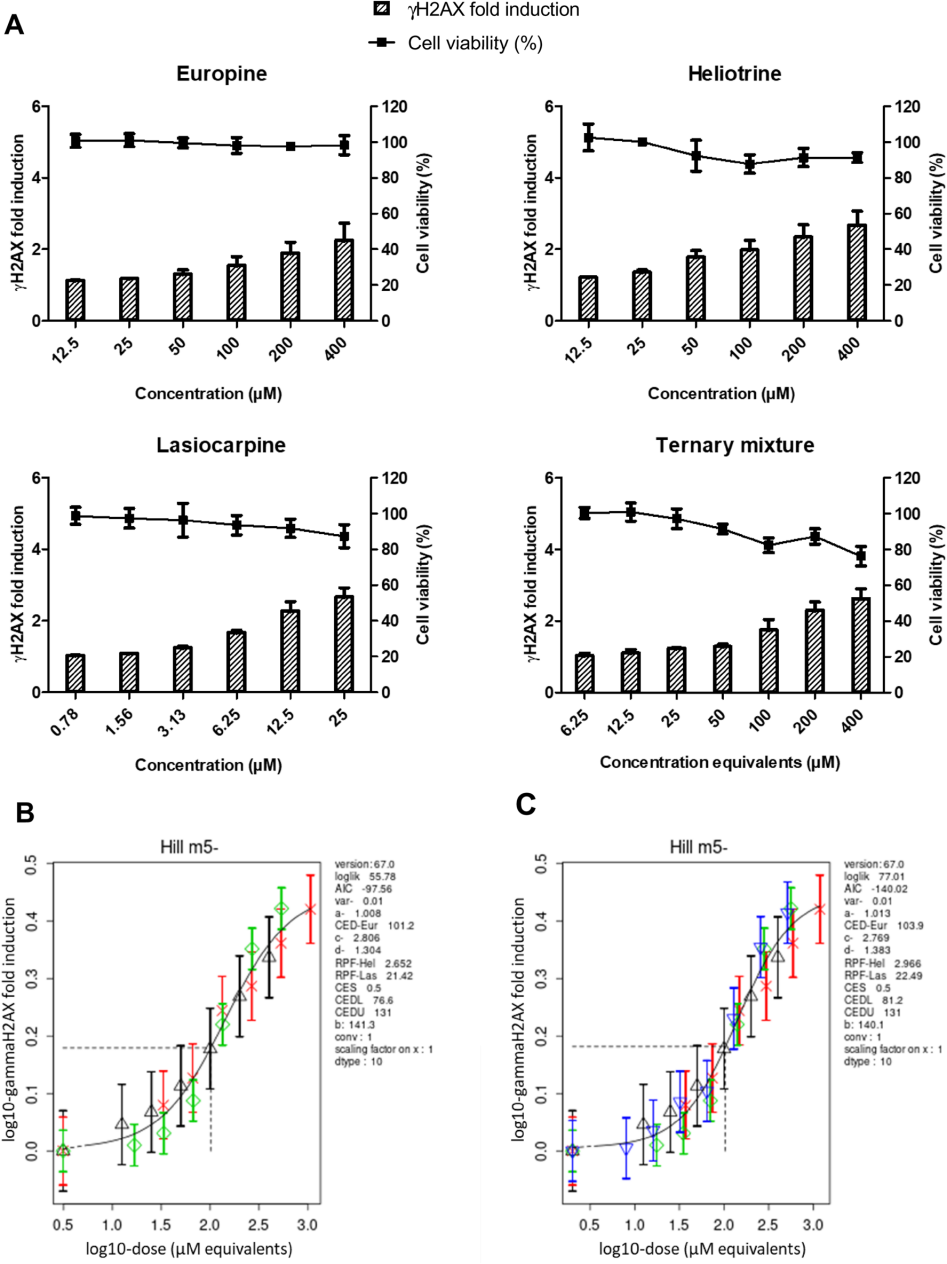
Concentration–response data and modelling for the PAs europine, heliotrine, and lasiocarpine and a ternary mixture thereof. **A** HepaRG cells were exposed to increasing concentrations of europine, heliotrine, and lasiocarpine and a ternary mixture as referred in Supplementary Table 5. After 24 h, cells were subjected to the WST-1 and γH2AX assays. **B**, **C** Concentration–response modelling of γH2AX induction data was performed as described in the materials and methods section using PROAST software. In the right-hand-side legend of the plots, a number of PROAST annotations and corresponding values are given that are related to the fitted model (including RPFs); for description of the annotations, see Supplementary Fig. 3. The values at the *x*-axis are concentration equivalents of the reference PA, europine. Green diamonds represent europine, red crosses heliotrine, black triangles lasiocarpine, and blue triangles the europine–heliotrine–lasiocarpine ternary mixture. The obtained curves represent the four-parameter Hill model. Concentration–response modelling and analysis for the single compounds (**B**) and the single compounds plus their mixture (**C**) show an overall fit of the concentration–response curves, pointing to dose addition as further described in the text

Europine was found to be the least potent of the three studied PAs, which was used as reference PA for the calculation of the RPFs of heliotrine and lasiocarpine (RPF for europine is set to 1). RPFs obtained for heliotrine and lasiocarpine using the exponential model were highly similar to those obtained with the Hill model. The RPFs determined applying the Hill model (RPF europine 1; RPF heliotrine 1.2; RPF lasiocarpine 16) were used to determine equipotent concentrations applied in the subsequent ternary mixture experiment (Supplementary Table 5). For this, increasing concentrations of the three individual PAs and the ternary mixture were tested side by side in the γH2AX assay. A WST-1 assay was also performed to examine for the occurrence of possible cytotoxic effects. The individual PAs and the ternary mixture showed a concentration-dependent increase in the induction of γH2AX with no to limited effects on cell viability (Fig. 4A).

Subsequently, PROAST was used for modelling the γH2AX data first excluding the mixture data (Fig. 4B) and then including the mixture data (Fig. 4C) to analyse whether the mixture effects follow dose addition principles (analysis with Hill models are shown, results obtained with the exponential models were very similar (data not shown)). If dose addition applies for the mixture, the response data points of the mixture fit with those of the single compounds (Fig. 4C), leading to an overall curve fit which is comparable to the curve fit derived only by fitting the concentrations-response data of the single compounds (Fig. 4B). To assess whether dose addition applies, RPFs and corresponding confidence intervals (CIs) obtained by modelling the single compounds and by modelling the single compounds plus the mixture were compared, showing only a slight change for the RPFs. The RPF of heliotrine and lasiocarpine shifts from 2.65 (CI 2.06–3.42) and 21.42 (CI 16.8–27.2) when only single compound data are taken into account to 2.97 (CI 2.35–3.75) and 22.49 (CI 17.8–28.4) if the mixture data are also considered (Fig. 4B, C). This indicates that the curve fit does not change significantly if the mixture is considered in the modelling and the confidence intervals of the RPFs overlap, which is in line with the assumption of dose addition for mixtures of PAs (Lichtenstein et al. 2020).

### γH2AX activity and PA content of *H. europaeum* extract fractions

**Fig. 5:**
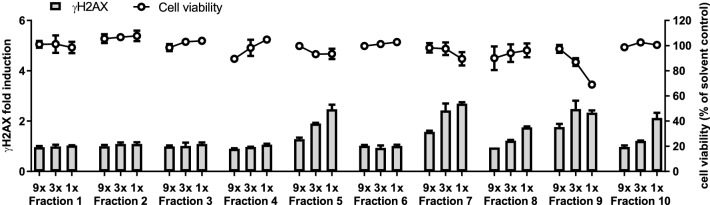
Effects of different dilutions of the 10 fractions of reduced *H. europaeum* extract on viability of HepaRG cells (circles, right *Y-*axes) and γH2AX induction (bars, left *Y*-axes). For each condition, mean values (± SD) from two independent experiments are presented

Since the γH2AX signal of the plant extracts was found to be higher than the signal of the artificial mixtures (Fig. 3), the *H. europaeum* extract was fractionated to allow a more focussed search for other genotoxic PAs. A total of 10 fractions were prepared using a Diode Array HPLC system and analysed using LC–MS/MS for the presence of the 35 PAs (Table 2) as well as for the necine base content (Table 3). In addition, the fractions were tested in the HepaRG/γH2AX assay. Induction of γH2AX was observed for fractions 5, 7, 8, 9, and 10 without affecting cell viability, except for undiluted fraction 9, which was slightly cytotoxic (Fig. 5). When combining the LC–MS/MS data with the γH2AX assay data, the γH2AX signal of fractions 5, 7 and 9 can be attributed (or at least for the major part) to the presence of europine, heliotrine, and lasiocarpine, respectively. In fractions 8 and 10, only small amounts of these three active PAs were detected (heliotrine and europine in fraction 8, and europine and lasiocarpine in fraction 10), suggesting that other compounds contribute substantially to the γH2AX signal induced by these fractions. The necine base analysis indicated the presence of heliotridine base mono or diester compounds in these fractions, suggesting that these PAs may be responsible for this γH2AX activity (Table 3). Retronecine-type PAs were not detected in any of the fractions.

**Table 2:** PA content (mg/l) of the reduced DMSO extracts of *H. europaeum* fractions

	Total 35 PAs	Major 6 PAs^a^	Minor 29 PAs^b^	Echinatine	Echinatine N-oxide	Rinderine	Rinderine N-oxide	Heliotrine	Heliotrine N-oxide	Europine	Europine N-oxide	Heliosupine	Heliosupine N-oxide	Lasiocarpine	Lasiocarpine N-oxide
Fr 1	ND	ND	ND	ND	ND	ND	ND	ND	ND	ND	ND	ND	ND	ND	ND
Fr 2	37	37	ND	ND	ND	ND	ND	ND	11	17	9	ND	ND	ND	ND
Fr 3	15,583	15,395	188	ND	20	ND	168	ND	ND	13	15,382	ND	ND	ND	ND
Fr 4	110	107	3	ND	ND	ND	3	4	25	27	42	ND	ND	ND	9
Fr 5	25,199	25,139	60	17	ND	43	ND	10	6960	17,867	293	ND	ND	ND	8
Fr 6	152	26	126	ND	ND	109	ND	ND	ND	23	3	ND	17	ND	ND
Fr 7	10,568	10,568	ND	ND	ND	ND	ND	9625	165	166	11	ND	ND	ND	601
Fr 8	74	32	42	ND	ND	ND	ND	10	3	13	ND	42	ND	ND	6
Fr 9	1826	1826	ND	ND	ND	ND	ND	3	ND	72	ND	ND	ND	1691	61
Fr 10	28	28	ND	ND	ND	ND	ND	ND	ND	4	ND	ND	ND	24	ND

*ND* not detectable

^a^Major 6 PAs: heliotrine, heliotrine N-oxide, europine, europine N-oxide, lasiocarpine, lasiocarpine N-oxide

^b^Minor PAs detected: echinatine, echinatine N-oxide, rinderine, rinderine N-oxide, heliosupine, heliosupine N-oxide

**Table 3:** PA necine content (mg/l) of the reduced DMSO extracts of *H. europaeum* fractions

	Total necine base	Retronecine	Retronecine N-oxide	Heliotridine	Heliotridine N-oxide
Fr 1	2.3	ND	ND	2.0	0.4
Fr 2	124	ND	ND	14	111
Fr 3	22,378	ND	ND	101	22,277
Fr 4	295	ND	ND	219	76
Fr 5	30,946	ND	ND	21,491	9456
Fr 6	260	ND	ND	203	57
Fr 7	15,894	ND	ND	14,946	948
Fr 8	214	ND	ND	98	115
Fr 9	1760	ND	ND	1678	81
Fr 10	314	ND	ND	297	17

The concentrations have been calculated based on the predominant known PA in each fraction

*ND* not detectable

**Fig. 6:**
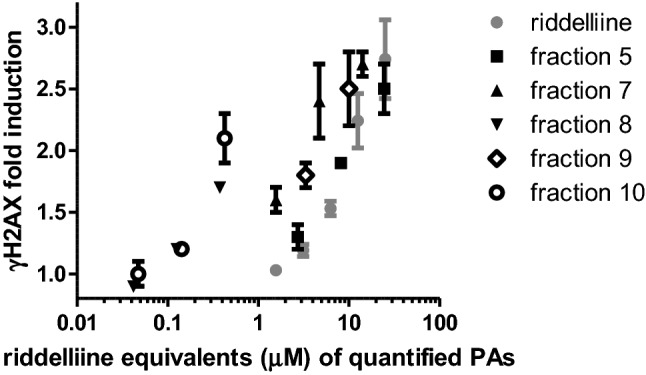
γH2AX activity of active fractions of reduced *H. europaeum* extract (Fig. 5) expressed in riddelliine equivalents of quantified PAs (Table 2) compared to the concentration–response curve for riddelliine-induced γH2AX activity. Riddelliine data were taken from Louisse et al. (2019). The highest concentration of fraction 9 was excluded because of substantial cytotoxicity (see Fig. 5)

To obtain more insight into the contribution of the quantified PAs in the active fractions to the γH2AX activity, we expressed the concentrations of these PAs (Table 2) in the active fractions (5, 7, 8, 9 and 10) in riddelliine equivalents, using RPF values obtained in our previous study (Louisse et al. 2019), i.e. assuming no activity of echinatine and rinderine, an RPF of 0.09 for heliotrine and europine, an RPF of 0.65 for heliosupine and an RPF of 1.1 for lasiocarpine. Figure 6 presents the γH2AX-response of these fractions based on the calculated riddelliine equivalents together with the concentration–response curve of riddelliine that was taken from our previous study (Louisse et al. 2019). The figure shows that the concentration–response data of fraction 5 overlap with the riddelliine curve, suggesting that no other PA than the ones quantified, substantially contributes to the γH2AX-response of that fraction. The figure also shows that, as already indicated above, especially fraction 8 and 10 contain a substantial amount of γH2AX activity that is not directed to the quantified PAs. In addition, for fractions 7 and 9, it may be expected that other PAs than the ones quantified contribute to the γH2AX activity (Fig. 6). From this analysis, it can be concluded that fractions 7–10 may contain bioactive PAs not present in the list of 35 PAs considered by the European Commission.

### Identification of unknown PAs

Since fractions 7, 8, 9 and 10 were considered to contain PAs responsible for γH2AX activity not explained by the known PAs (LC–MS/MS analysis for 35 PAs), the 10 *H. europaeum* fractions were analysed for the presence of possible other PAs using full scan LC–Orbitrap-MS. Chromatographic conditions very similar to the LC–MS/MS measurements were used and ions were measured in the m/z 200–550 range (ESI+). For processing of the full scan data, Compound Discoverer software was used and several settings/criteria were applied to pinpoint the analysis and identification towards PAs (see for details the materials and methods section). Analysis of the vDIA and ddMS^2^ data resulted in a list of 120 potential PAs present in one or more fractions (Supplementary Table 6). On the basis of their elementary composition, fragmentation spectrum and retention time, 47 could be annotated as monoester derivatives of heliotridine or heliotridine N-oxide, and 25 as diester derivatives. The other 48 compounds most likely contain a platynecine base and are considered not toxicologically active. Furthermore, of the 72 mono and diester heliotridine analogues 31 were identified as N-oxides and thus not expected to be active in the HepaRG/γH2AX assay. Applying a cut off level for individual compounds (0.1% of the combined peak area) further reduced the number of potentially relevant analogues to 13 heliotridine monoesters, including echinatine, rinderine, heliotrine and europine, and 8 heliotridine diesters, including heliosupine and lasiocarpine (Table 4). Based on the obtained insights into the structure-related potencies of PAs in the γH2AX assay (Louisse et al. 2019), the detected monoesters are expected to be moderately active in the γH2AX assay (i.e. expected to have a similar activity as heliotrine and europine) and the diesters are expected to be highly active in the γH2AX assay (i.e. expected to have similar activity as lasiocarpine). More details on these PAs are presented in Table 4.

**Table 4:** PAs (tentatively) identified in DMSO extracts of *H. europaeum* fractions by LC–Orbitrap-MS

Elementary composition	Detected mass (amu)	Deviation (ppm)	RT (min)	Annotation	Annotation level^a^	Putative activity	Relative intensity^b^	Area, *1e7							
								Fr 4	Fr 5	Fr 6	Fr 7	Fr 8	Fr 9	Fr 10	Total
C_13_H_19_NO_4_	253.1310	− 1.47	8.59	Hydroxyangeloyl heliotridine (or isomer)	3	Medium	0.15%				11.6				11.6
C_15_H_25_NO_4_	283.1782	− 0.48	7.42	Supinine	2	Medium	0.11%			8.6					8.6
C_15_H_25_NO_5_	299.1728	− 1.58	6.82	Echinatine	1	Medium	0.13%		10.0						10.0
C_15_H_25_NO_5_	299.1728	− 1.54	6.99	Rinderine	1	Medium	1.17%		25.5	63.9					89.5
C_15_H_25_NO_6_	315.1676	− 1.87	4.92	5′− Hydroxyrinderine	2	Medium	0.68%	52.0							52.0
C_16_H_27_NO_4_	297.1936	− 1.29	9.62	Heleurine	2	Medium	0.95%					72.6			72.6
C_16_H_27_NO_5_	313.1881	− 2.77	8.61	Heliotrine	1	Medium	41.6%				3170	7.4			3177
C_16_H_27_NO_6_	329.1843	1.35	6.40	Europine isomer	3	Medium	0.27%		20.9						20.9
C_16_H_27_NO_6_	329.1830	− 2.66	6.58	Europine	1	Medium	37.8%	6.5	2790	11.9	70.2	6.1	19.0		2900
C_16_H_27_NO_6_	329.1839	0.09	6.81	Europine isomer	3	Medium	0.87%		65.8						65.8
C_16_H_27_NO_6_	329.1831	− 2.11	7.91	Europine isomer	3	Medium	0.15%			11.3					11.3
C_16_H_27_NO_7_	345.1782	− 1.67	5.85	Hydroxyeuropine isomer	3	Medium	0.11%		8.0						8.0
C_18_H_25_NO_5_	335.1720	− 2.60	12.12	Heliotridine 1,7− diester	3	High	0.39%							29.7	29.7
C_18_H_29_NO_7_	371.1938	− 1.69	8.59	5′− Acetyleuropine	2	Medium	0.68%				52.1				52.1
C_18_H_29_NO_7_	371.1937	− 1.87	8.85	7− Acetyleuropine (or isomer)	2	Medium	0.39%				29.9				29.9
C_20_H_31_NO_7_	397.2100	− 0.10	10.79	Heliosupine	1	High	0.36%					27.2			27.2
C_21_H_33_NO_6_	395.2311	0.81	12.38	7− Angeloylheliotrine	2	High	0.15%							11.2	9.2
C_21_H_33_NO_7_	411.2244	− 3.17	11.45	7− Tigloyleuropine	2	High	1.19%						90.8		90.8
C_21_H_33_NO_7_	411.2245	− 3.02	11.54	Lasiocarpine	1	High	10.2%						770	6.5	777
C_23_H_35_NO_8_	453.2369	1.38	11.86	5′− Acetyl− 7− tigloyleuropine	2	High	0.17%							13.1	13.1
C_23_H_35_NO_8_	453.2351	− 2.54	12.12	5′− Acetyllasiocarpine	2	High	2.48%							189	189
						Total area, *1e7		58.5	2920	95.7	3330	113	880	250	7650
						% of total area		0.8%	38.1%	1.3%	43.6%	1.5%	11.5%	3.3%	

Note: Only heliotridine mono- and diesters were considered

^a^Annotation levels: 1: authentic standard available; 2: literature data available, known component; 3: tentative assignment based on elementary composition and mass fragmentation spectrum

^b^Cutoff level for reporting compound: peak area: 0.1% of total peak area. Fraction 1–3 did not contain compounds above the cutoff level

**Fig. 7:**
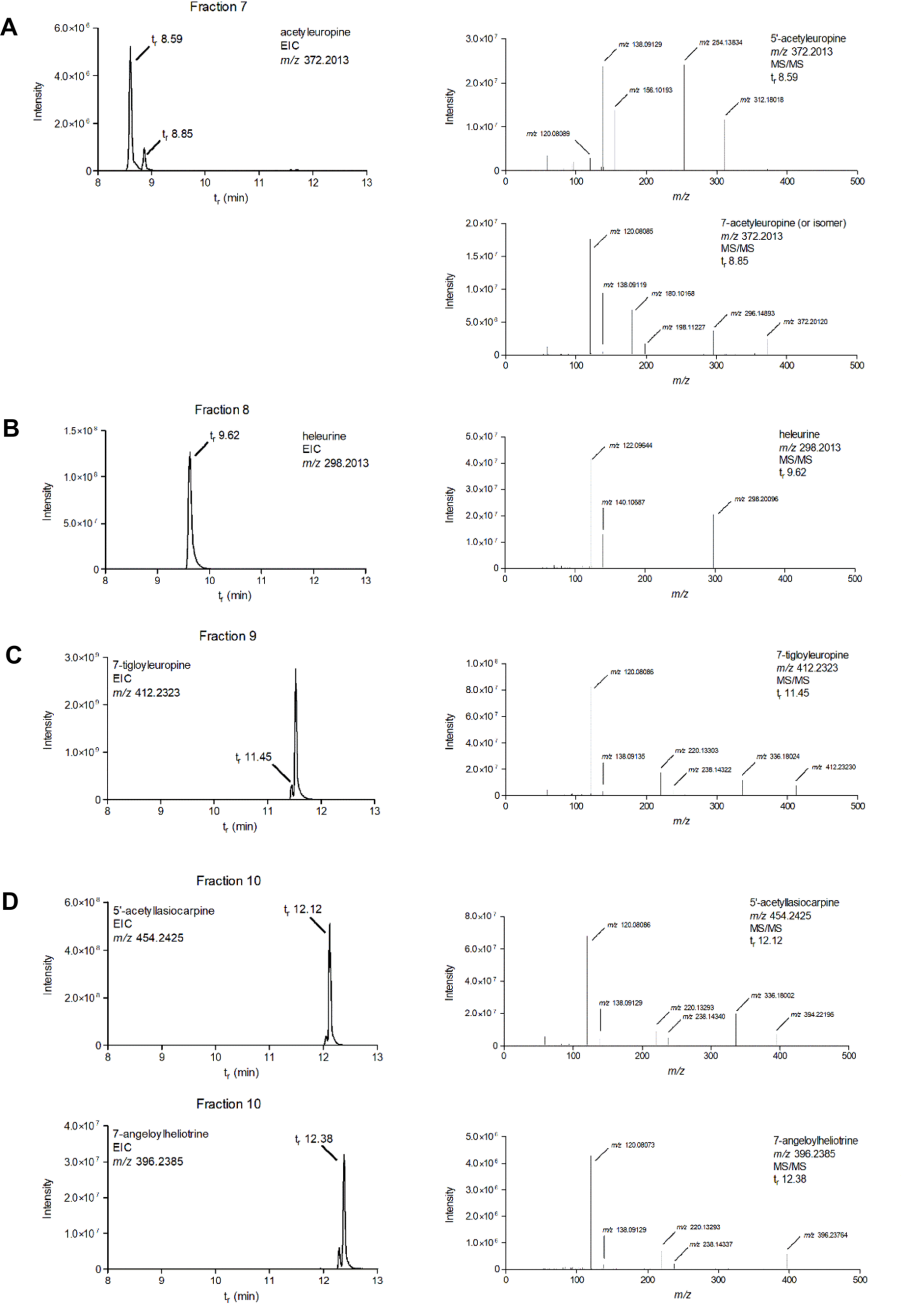
LC–Orbitrap-MS chromatograms and mass spectra of fractions 7–10 of the reduced extract of *H. europaeum*. **A** Fraction 7: 5′-acetyleuropine + 7-acetyleuropine. **B** Fraction 8: heleurine. **C** Fraction 9: 7-tigloyleuropine. **D** Fraction 10: 7-angeloylheliotrine + 5′-acetyllasiocarpine

As indicated in Table 4, particularly fraction 7 contains several potentially active PAs. Besides heliotrine, this fraction contains some europine. Fraction 7 also contains probably a hydroxyangeloyl heliotridine analogue and two acetyl derivatives of europine, one of which can be identified as 5′-acetyleuropine (Fig. 7A). This compound has been reported as a constituent of *H. europaeum* as well as of several other *Heliotropium* species (El-Shazly and Wink 2014; Mädge et al. 2020; Shimshoni et al. 2015, 2021). Based on its fragmentation spectrum, the other acetyl derivative is probably 7-acetyleuropine, which has been reported from *H. bovei*, a related *Heliotropium* species (El-Shazly and Wink 2014).

Fraction 8 contains less (active) PAs (Table 4) than fraction 7, as also indicated by the lower γH2AX response (Fig. 5). Besides limited amounts of heliotrine and europine, it contains the open-diester heliosupine. LC–Orbitrap analysis further revealed the presence of heleurine (Fig. 7B). Heleurine has been reported as a constituent of *H. europaeum* (El-Shazly and Wink 2014).

Fraction 9 contains, besides large amounts of lasiocarpine and a very limited amount of europine, a structural isomer of lasiocarpine, which can be tentatively identified as 7-tigloyleuropine (Fig. 7C). Based on the peak areas the concentration of the tigloyl ester is about 12% of that of lasiocarpine. 7-Tigloyleuropine as a constituent of *H. europaeum* has been reported by Shimshoni et al. (2015, 2021).

Five main peaks were found in fraction 10 that after analysis of the mass spectrum could be attributed to the open-diester PAs lasiocarpine and 5′-acetyllasiocarpine, 5′-acetyl-7-tigloyleuropine, 7-angeloylheliotrine and an unidentified heliotridine diester (Table 4; Fig. 7D). In particular 5′-acetyllasiocarpine is present in a relatively high concentration [based on the TIC of combined fractions it could represent 2.5% of the compounds potentially active in the γH2AX assay (Table 4)]. 5′-Acetyllasiocarpine and 7-angeloylheliotrine are known PAs of *H. europaeum* and related species (El-Shazly and Wink 2014; Mädge et al. 2020; Shimshoni et al. 2015, 2021).

Altogether, from the analyses of the full scan LC–Orbitrap-MS data of fractions 7–10 and information on reported PAs in *Heliotropium* species from the literature, it can be concluded that potential active PAs present in the reduced *H. europaeum* extract, not part of the list of 35 PAs considered by the European Commission, include heleurine, 5′-acetyleuropine, 7-acetyleuropine, 7-tigloyleuropine, 5′-acetyllasiocarpine and 7-angeloylheliotrine. LC–Orbitrap-MS chromatograms and fragmentation spectra of the detected bioactive PAs, reported before to be present in *Heliotropium* species, but not included in the list of 35 PAs considered by the European Commission are shown in Fig. 7. It must be noted that besides these PAs, also other active PAs seem to be present that could not be identified in more detail (e.g. several analogues of europine and especially the heliotridine diester in fraction 10).

## Discussion

The aim of the present study was to assess whether a bioassay-directed analysis approach can identify relevant PAs not yet included in monitoring programmes. To that end, extracts of *H. europaeum* and *H. popovii* were analysed for the presence of 35 known PAs as included in monitoring programmes and for γH2AX activity. Comparison of γH2AX activity of the extracts with that of an artificial mixture of the quantified known PAs suggested that other PAs present in the plant extracts contributed to the γH2AX activity. Fractionation of the *H. europaeum* extract, followed by quantification of the known PAs, the necine base content and the γH2AX activity pointed to two fractions with a large amount and two fractions with a lesser amount of unexplained γH2AX activity. Necine base analysis showed that only PAs with a heliotridine base were present in these fractions. By applying LC–Orbitrap-MS analysis and Compound Discoverer software on these fractions, we tentatively identified heleurine, 5′-acetyleuropine, 7-acetyleuropine, 7-tigloyleuropine, 5′-acetyllasiocarpine, and 7-angeloylheliotrine as PAs present in *H. europaeum*, that likely contribute to the total genotoxic activity of the *H. europaeum* extract.

Bioassay-directed identification approaches have been applied to identify chemicals with divergent bioactivity, such as endocrine activity [e.g. estrogenic activity (Nielen et al. 2004) and anti-androgenic activity (Rostkowski et al. 2011)], and antimicrobial activity (Wegh et al. 2017). Furthermore, application of a strategy based on broad screening and bioassay-directed identification with liquid chromatography high-resolution mass spectrometry (LC–HRMS) has been proposed to prevent intoxications and identify toxins and toxicants relevant for food and feed safety (Gerssen et al. 2019). Specific examples on the application of bioassay-directed identification approaches in food and feed include the identification of brominated dioxins in the feed additive choline chloride (Traag et al. 2009), natural aryl hydrocarbon receptor (AhR) agonists in marmalade (Van Ede et al. 2008), and the potent sulfotransferase inhibitor nevadensin in basil (Alhusainy et al. 2010). Bioassay-directed identification strategies to identify genotoxic chemicals have been applied for coastal sediments (Fernandez et al. 1992), surface water (Grifoll et al. 1992), urban airborne particulate matter (Casellas et al. 1995), bioremediated soils (Brooks et al. 1998), and to identify mutagenic nitrogenous disinfection by-products of advanced oxidation drinking water treatment (Vughs et al. 2018). These studies mainly applied the Ames test for mutagenicity assessment. To the best of our knowledge, no examples on bioassay-directed approaches to identify genotoxic chemicals in food or feed have been described in the literature. The present study provides the first example of application of the HepaRG/γH2AX assay to identify relevant genotoxic PAs in *H. europaeum*, a noxious weed that can contaminate both food and feed.

The 6 PAs tentatively identified have been previously reported to be present in several *Heliotropium* species, including *H. europaeum* (El-Shazly and Wink 2014; Mädge et al. 2020; Shimshoni et al. 2015, 2021). Unfortunately, these PAs are not commercially available, so to confirm the identity of these PAs, standards should become available, or they should be isolated from *H. europaeum* extracts, to have their identities confirmed by NMR and their toxic potencies determined in the HepaRG/γH2AX assay. Furthermore, no data on the genotoxicity of these PAs are available in the literature. Considering reported genotoxicity of other PAs and related information on structure–activity relationships, it is expected that the open-diester PAs 7-acetyleuropine, 7-tigloyleuropine, 5′-acetyllasiocarpine and 7-angeloylheliotrine are relatively potent genotoxicants, as previously tested open-diester PAs belong to the group of most potent PAs in the HepaRG/γH2AX assay (Louisse et al. 2019). Heleurine and 5′-acetyleuropine are monoester PAs, which are expected to have lower genotoxic potencies, based on the results of our earlier studies testing 37 PAs in the HepaRG/γH2AX assay (Louisse et al. 2019). Even though direct confirmations of the tentatively identified PAs could not be made in the present study, the previous reporting of these PAs in *H. europaeum* and other *Heliotropium* species combined with knowledge on structure–activity relationships of PAs regarding their (geno)toxic potential provides confidence in the relevance of our findings. It must be noted that besides these 6 PAs, and the 6 major PAs for which standards were available, at least 6 other PAs may be of relevance (annotation level 3 Table 4), especially those for which a medium to high level of toxicity is expected. Further identification efforts of such PAs could be considered if still a large portion of non-explained activity remains, when a quantitative assessment of the contribution of the 6 PAs could be made to the total response of the *H. europaeum* extract in the HepaRG/γH2AX assay. Although PAs are generally considered as the genotoxic constituents of a large number of species from Asteraceae, Boraginaceae and Fabaceae, it cannot be ruled out that other phytotoxins in the *Heliotropium* extracts have contributed to the total genotoxicity response measured with the HepaRG/γH2AX assay, since the extraction method used is not specific for PAs. On the other hand, an extract of chamomile (*M. recutita*, Asteraceae), which was included in the study as a non-PA-containing reference extract, didn’t result in a signal in the γH2AX assay (Fig. 2). Nevertheless, the genotoxicity of tentatively identified bioactive PAs needs to be confirmed and quantified in the γH2AX assay using authentic reference standards to determine whether the non-explained genotoxic activity in the *Heliotropium* extracts is only attributed to these PAs.

*Heliotropium europaeum* was selected in this study, because it belongs to a genus of which various species have been involved in serious episodes of intoxications related to feed as well as food (Shimshoni et al. 2015; Wiedenfeld 2011). Recently, *Heliotropium* species have been implicated as the main PA-containing contaminants in oregano herbs and cumin spice (Picron et al. 2021), resulting in a multitude of RASFF notifications (EU 2021). The methodology can also be applied to other plant species, that are potential contaminants of foods, such as *Echium, Eupatorium* and *Symphytum* species. Like *Heliotropium* species, these plants contain mostly open chain mono and diesters (El Shazly and Wink 2014, Mägde et al. 2020). *Senecio* and *Jacobaea* species should also be considered as these are well known for their weed potential and containing a wide range of macrocyclic PAs that could significantly add to the genotoxic potential of these plants (Jung et al. 2020).

Altogether, the present study shows how a bioassay-directed analysis approach allowed to tentatively identify heleurine, 5′-acetyleuropine, 7-acetyleuropine, 7-tigloyleuropine, 5′-acetyllasiocarpine and 7-angeloylheliotrine as possible active PAs in *H. europaeum*. These PAs are not yet part of the list of 35 PAs considered by the European Commission as being relevant for monitoring in food and feed, and for which maximum levels in foodstuffs have been set. It is recommended to isolate and/or synthesise these PAs and assess their genotoxicity. When their genotoxicity is confirmed, these PAs can be considered relevant candidates to be included in monitoring programmes.

## Supplementary Information

Below is the link to the electronic supplementary material.

Supplementary file1 (PDF 93 KB)

Supplementary file2 (PDF 174 KB)

Supplementary file3 (PDF 159 KB)

Supplementary file4 (PDF 125 KB)

Supplementary file5 (PDF 136 KB)

Supplementary file6 (PDF 104 KB)

Supplementary file7 (PDF 244 KB)

Supplementary file8 (PDF 111 KB)

Supplementary file9 (PDF 422 KB)
