# Supplementary material for: Bioassay-directed analysis-based identification of relevant pyrrolizidine alkaloids
Source: Arch Toxicol. 2022 May 24;96(8):2299–317. doi: 10.1007/s00204-022-03308-z (PMC9217854; doi:10.1007/s00204-022-03308-z)
Supplement: Supplementary file 1 — Supplementary file1 (PDF 93 KB) [file 204_2022_3308_MOESM1_ESM.pdf]

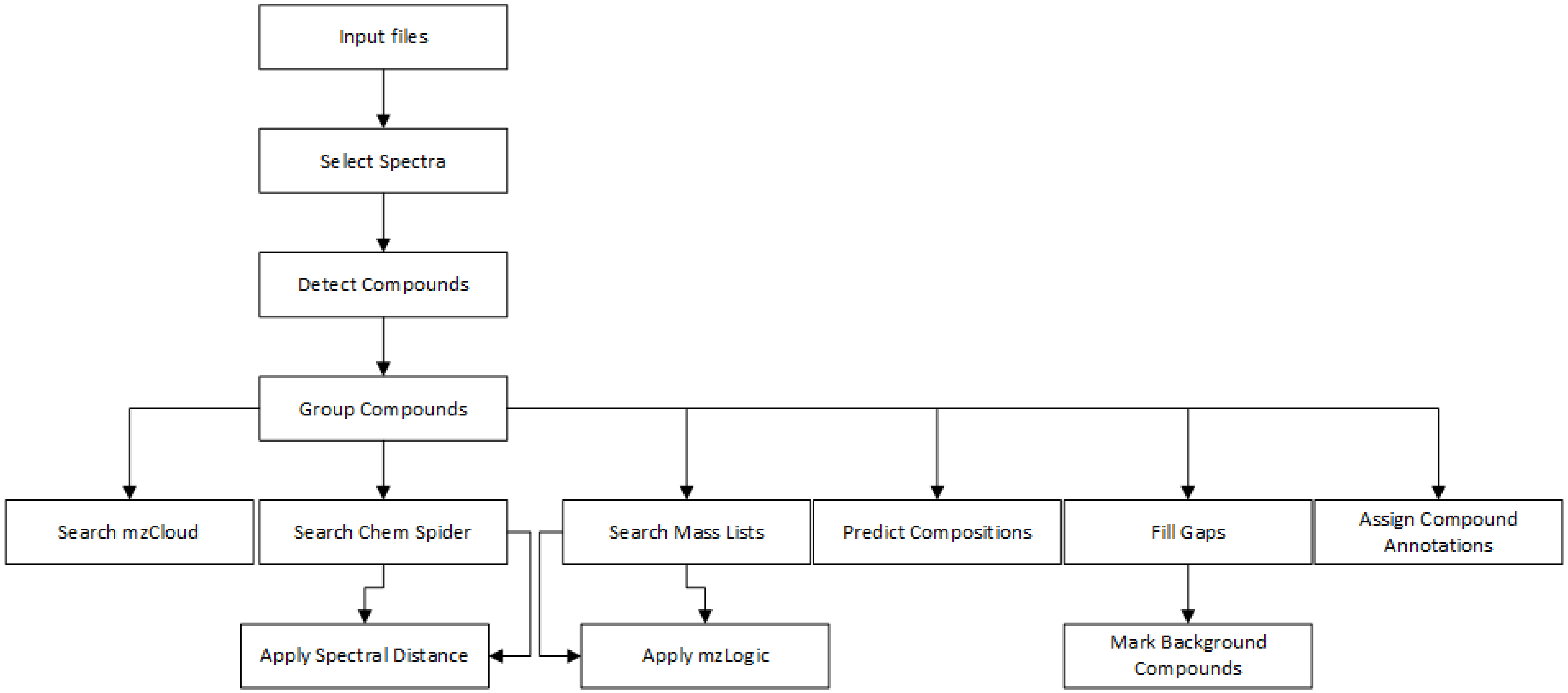

Supplementary Figure 1. Schematic overview of LC-Orbitrap-MS data processing performed using Thermo Scientific Compound Discoverer 3.1. More information is provided in section ‘Bioassay- directed identification’.
