# Supplementary material for: Bioassay-directed analysis-based identification of relevant pyrrolizidine alkaloids
Source: Arch Toxicol. 2022 May 24;96(8):2299–317. doi: 10.1007/s00204-022-03308-z (PMC9217854; doi:10.1007/s00204-022-03308-z)
Supplement: Supplementary file 2 — Supplementary file2 (PDF 174 KB) [file 204_2022_3308_MOESM2_ESM.pdf]

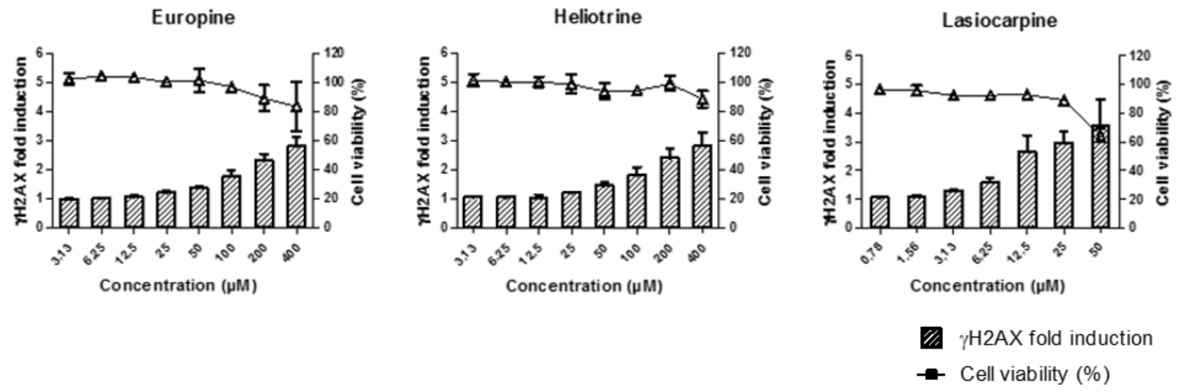


**Supplementary Figure 2.** Concentration-dependent effects of the PAs europine, heliotrine, and lasiocarpine on γH2AX induction (bars, left Y-axes) in, and cell viability (triangles, right Y-axes) of HepaRG cells. For each condition, mean values (± SD) from two independent experiments are presented.
