# Supplementary material for: Bioassay-directed analysis-based identification of relevant pyrrolizidine alkaloids
Source: Arch Toxicol. 2022 May 24;96(8):2299–317. doi: 10.1007/s00204-022-03308-z (PMC9217854; doi:10.1007/s00204-022-03308-z)
Supplement: Supplementary file 3 — Supplementary file3 (PDF 159 KB) [file 204_2022_3308_MOESM3_ESM.pdf]

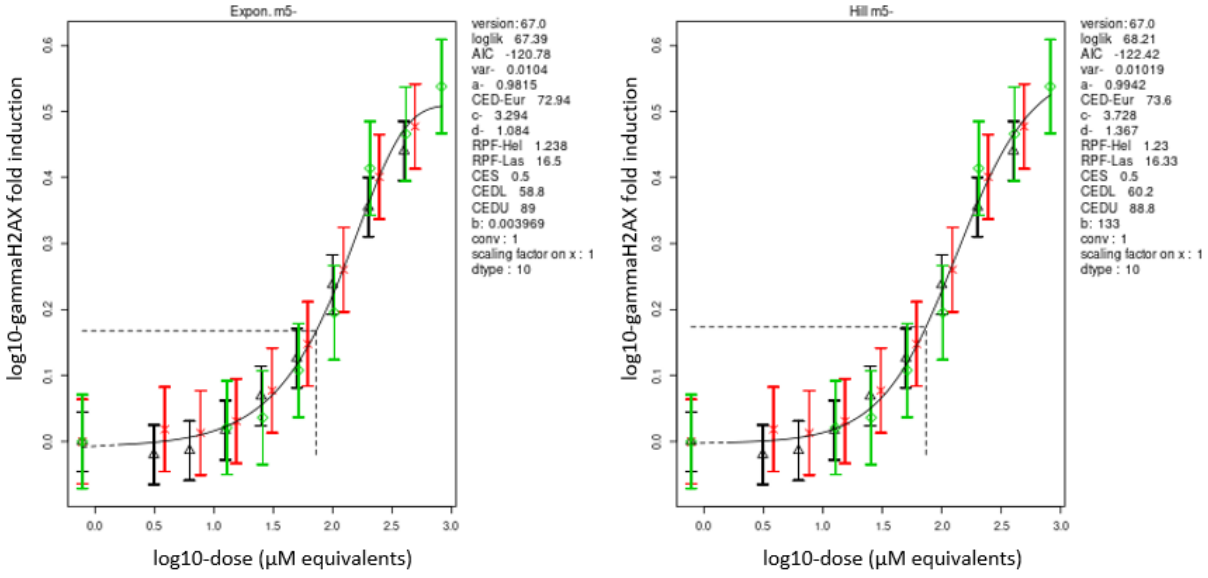

Supplementary Figure 3. Determination of preliminary RPFs for the individual PAs europine, heliotrine and lasiocarpine on the basis of BMD modelling of concentration-response data (γH2AX induction). The figure shows the output files generated by PROAST, presenting on the left the exponential model and on the right the Hill model, which were the two models demonstrating the best fit (lowest AIC value). The values at the x-axis represent concentration equivalents of europine (least potent PA). For both models the BMC (same as critical effect dose, CED) and the corresponding two-sided 90% BMC confidence interval (CI) given by the BMCL (lower bound of the BMC CI; same as CEDL) and the BMCU (upper bound of the BMC CI; same as CEDU) are presented. For calculation of RPFs, the BMC (or CED) of europine was used as reference (RPF=1). CED, CEDL, CEDU and RPF values were calculated for a critical effect size (CES) of 0.5, which corresponds to a benchmark response (BMR) of 50%. The symbols (green diamonds for europine, red crosses for heliotrine, black triangles for lasiocarpine) represent average values of two independent experiments; vertical lines (error bars) represent 90% CIs of the average values and the dashed horizontal and vertical lines indicate the BMR of 50% and the corresponding BMC, respectively; a,b,c,d represent values for fitted model parameters; for information on other abbreviations, the reader is referred to https://proastweb.rivm.nl.
