# Supplementary material for: Bioassay-directed analysis-based identification of relevant pyrrolizidine alkaloids
Source: Arch Toxicol. 2022 May 24;96(8):2299–317. doi: 10.1007/s00204-022-03308-z (PMC9217854; doi:10.1007/s00204-022-03308-z)
Supplement: Supplementary file 4 — Supplementary file4 (PDF 125 KB) [file 204_2022_3308_MOESM4_ESM.pdf]

Supplementary Table 1. Information on analytical standards used.

Name	CAS no	Purity (%)	Supplier	Article no
Echimidine	520-68-3	99.2	PhytoPlan	6278.95
Echimidine N-oxide	41093-89-4	97.0	PhytoPlan	6279.97
Echinatine	480-83-1	96.7	PhytoPlan	6295.95
Echinatine N-oxide	20267-93-0	97.3	PhytoPlan	6296.95
Europine (hydrochloride salt)	570-19-4	97.0	PhytoPlan	6214.97
Europine N-oxide	65582-53-8	99.7	PhytoPlan	6215.97
Heliosupine	32728-78-2	95.0	PhytoPlan	6297.95
Heliosupine N-oxide	31701-88-9	95.0	PhytoPlan	6297.95
Heliotrine	303-33-3	90.0	Latoxan	L6007
Heliotrine N-oxide	6209-65-0	97.0	PhytoPlan	6213.97
Indicine (hydrochloride salt)	1195140-94-3	98.3	PhytoLab	83234
Indicine N-oxide	41708-76-3	99.4	PhytoPlan	6217.96
Integerrimine	480-79-5	97.0	PhytoPlan	6283.97
Integerrimine N-oxide	85955-28-8	95.0	PhytoPlan	6284.95
Intermedine	10285-06-0	95.0	PhytoPlan	6274.95
Intermedine N-oxide	95462-14-9	95.0	PhytoPlan	6275.95
Lasiocarpine	303-34-5	95.0	PhytoLab	89726
Lasiocarpine N-oxide	127-30-0	96.7	PhytoPlan	6211.96
Lycopsamine	10285-07-1	97.5	PhytoPlan	6270.95
Lycopsamine N-oxide	95462-15-0	98.7	PhytoPlan	6271.95
Retrorsine	480-54-6	99.0	PhytoLab	89775
Retrorsine N-oxide	15503-86-3	96.0	PhytoPlan	6253.96
Rinderine	6029-84-1	95.0	PhytoPlan	6310.95
Rinderine N-oxide	137821-16-0	95.0	PhytoPlan	6311.95
Senecionine	130-01-8	99.0	PhytoPlan	6202.99
Senecionine N-oxide	13268-67-2	95.0	PhytoPlan	6252.95
Seneciphylline	480-81-9	99.6	PhytoLab	89275
Seneciphylline N-oxide	38710-26-8	99.6	PhytoLab	82632
Senecivernine	72755-25-0	99.0	PhytoPlan	6206.95
Senecivernine N-oxide	101687-28-9	99.5	PhytoPlan	6220.95
Senkirkine	2318-18-5	98.3	PhytoLab	89274
Spartioidine	520-59-2	95.1	PhytoPlan	6314.95
Spartioidine N-oxide	121123-61-3	95.0	In house synthesized	
Usaramine	15503-87-4	96.0	PhytoPlan	6315.96
Usaramine N-oxide	117020-54-9	96.0	PhytoPlan	6316.96
Dibutylheliotridine N-oxide-D2		95.2	Chiroblock	
Dibutylheliotridine-D2		96.2	Chiroblock	
Dibutylretronecine N-oxide-D2		95.0	Chiroblock	
Dibutylretronecine-D2		95.0	Chiroblock	
Heliotridine	250-63-8	97.0	PhytoPlan	6286.97
Heliotridine N-oxide		97.0	PhytoPlan	6287.97
Retronecine	480-85-3	95.0	PhytoPlan	6285.95
Retronecine N-oxide	6870-33-3	97.0	PhytoPlan	6285.97
