# Supplementary material for: Bioassay-directed analysis-based identification of relevant pyrrolizidine alkaloids
Source: Arch Toxicol. 2022 May 24;96(8):2299–317. doi: 10.1007/s00204-022-03308-z (PMC9217854; doi:10.1007/s00204-022-03308-z)
Supplement: Supplementary file 5 — Supplementary file5 (PDF 136 KB) [file 204_2022_3308_MOESM5_ESM.pdf]

Supplementary Table 2. Mass spectrometric settings for the analysis covering 35 PAs included in EU legislation.

**Pyrrolizidine alkaloid**	**Precursor ion (m/z)**	**Cone voltage (V)**	**Product ion 1 (m/z)**	**Col. energy 1 (eV)**	**Product ion 2 (m/z)**	**Col. energy 2 (eV)**	**Product ion 3 (m/z)**	**Col. energy 3 (eV)**	**Indicative RT (min)**
Intermedine	300.2	30	94	35	156	30	138	30	5.45*
Lycopsamine	300.2	30	94	35	156	30	138	30	5.50*
Indicine	300.2	30	94	35	156	30	138	30	5.50*
Echinatine	300.2	30	138	30	156	30	94	35	6.45
Rinderine	300.2	30	138	30	156	30	94	35	6.60
Heliotrine	314.2	30	138	25	156	25	94	35	8.10
Intermedine N-oxide	316.2	30	94	40	172	30	111	40	3.40*
Lycosamine N-oxide	316.2	30	94	40	172	30	111	40	3.45*
Indicine N-oxide	316.2	30	94	40	172	30	111	40	3.55
Echinatine N-oxide	316.2	30	111	40	172	30	94	40	3.75
Rinderine N-oxide	316.2	30	111	40	172	30	94	40	3.85
Europine	330.2	30	94	35	138	30	156	30	6.15
Heliotrine N-oxide	330.2	30	111	35	172	25	94	40	5.50
Spartioidine	334.2	40	120	30	138	30	94	40	8.95
Seneciphylline	334.2	40	120	30	138	30	94	40	9.25
Integerrimine	336.2	40	94	40	120	30	138	30	9.95
Senecionine	336.2	40	94	40	120	30	138	30	10.25
Senecivernine	336.2	40	94	40	120	30	138	30	10.40
Europine N-oxide	346.2	30	172	30	111	40	256	25	3.55
Spartioidine N-oxide	350.2	40	94	40	120	30	118	30	5.70
Seneciphylline N-oxide	350.2	40	94	40	138	30	118	30	5.80
Usaramine	352.2	40	94	40	120	30	138	30	8.30
Retrorsine	352.2	40	94	40	120	30	138	30	8.55
Integerrimine N-oxide	352.2	40	94	40	120	30	136	30	6.50
Senecionine N-oxide	352.2	40	94	40	120	30	136	30	6.70*
Senecivernine N-oxide	352.2	40	94	40	120	30	136	30	6.75*
Senkirkine	366.2	30	122	30	168	25	150	25	7.00
Usaramine N-oxide	368.2	40	94	40	120	30	119	30	5.25
Retrorsine N-oxide	368.2	40	94	40	120	30	119	30	5.40
Heliosupine	398.2	30	120	25	220	20	336	20	10.40
Echimidine	398.2	30	120	25	220	20	83	25	10.55
Lasiocarpine	412.2	30	120	25	220	20	336	20	11.25
Heliosupine N-oxide	414.2	30	94	30	254	30	138	30	7.10
Echimidine N-oxide	414.2	30	254	30	352	25	94	40	7.40
Lasiocarpine N-oxide	428.2	30	138	30	254	25	94	40	8.25

*: isomeric compounds that are not fully chromatographically separated.
