# Supplementary material for: Bioassay-directed analysis-based identification of relevant pyrrolizidine alkaloids
Source: Arch Toxicol. 2022 May 24;96(8):2299–317. doi: 10.1007/s00204-022-03308-z (PMC9217854; doi:10.1007/s00204-022-03308-z)
Supplement: Supplementary file 6 — Supplementary file6 (PDF 104 KB) [file 204_2022_3308_MOESM6_ESM.pdf]

Supplementary Table 3. Mass spectrometric settings for the analysis of the PA necine bases.

Necine base	Precursor ion (m/z)	Cone voltage (V)	Product ion 1 (m/z)	Col. energy 1 (eV)	Product ion 2 (m/z)	Col. energy 2 (eV)	Product ion 3 (m/z)	Col. energy 3 (eV)	Indicative RT (min)
Retronecine	156.2	30	80	25	94	20	108	20	3.75
Heliotridine	156.2	30	80	25	94	20	108	20	4.20
Retronecine-d2	158.2	30	80	25	96	20	108	20	3.75
Heliotridine-d2	158.2	30	80	25	96	20	108	20	4.20
Retronecine N-oxide	172.2	30	80	40	94	25	111	30	3.30
Heliotridine N-oxide	172.2	30	80	40	94	25	111	30	3.55
Retronecine N-oxide-d2	174.2	30	80	40	96	25	113	30	3.30
Heliotridine N-oxide-d2	174.2	30	80	40	96	25	113	30	3.55
