# Supplementary material for: Bioassay-directed analysis-based identification of relevant pyrrolizidine alkaloids
Source: Arch Toxicol. 2022 May 24;96(8):2299–317. doi: 10.1007/s00204-022-03308-z (PMC9217854; doi:10.1007/s00204-022-03308-z)
Supplement: Supplementary file 7 — Supplementary file7 (PDF 244 KB) [file 204_2022_3308_MOESM7_ESM.pdf]

Supplementary Table 4. Mass list Open chain pyrrolizidine alkaloids (n=296).

ID	Name	Formula	Molecular Weight (Da)
1	supinidine-type retronecine/heliotridine	C8 H13 N O	139.09971
2	retronecine/heliotridine	C8 H13 N O2	155.09463
3	N-oxide/hydroxy retronecine/heliotridine	C8 H13 N O3	171.08954
4	dihdyroxy retronecine/heliotridine	C8 H13 N O4	187.08446
5	trihydroxy retronecine/heliotridine	C8 H13 N O5	203.07937
6	supinidine-type platynecine	C8 H15 N O	141.11536
7	platynecine	C8 H15 N O2	157.11028
8	N-oxide/hydroxy platynecine	C8 H15 N O3	173.10519
9	dihdyroxy platynecine	C8 H15 N O4	189.10011
10	trihydroxy platynecine	C8 H15 N O5	205.09502
11	acetyl supinidine-type retronecine/heliotridine	C10 H15 N O2	181.11028
12	acetyl retronecine/heliotridine	C10 H15 N O3	197.10519
13	acetyl N-oxide/hydroxy retronecine/heliotridine	C10 H15 N O4	213.10011
14	acetyl dihydroxy retronecine/heliotridine	C10 H15 N O5	229.09502
15	acetyl trihydroxy retronecine/heliotridine	C10 H15 N O6	245.08994
16	acetyl supinidine-type platynecine	C10 H17 N O2	183.12593
17	acetyl platynecine	C10 H17 N O3	199.12084
18	acetyl N-oxide/hydroxy platynecine	C10 H17 N O4	215.11576
19	acetyl dihydroxy platynecine	C10 H17 N O5	231.11067
20	acetyl trihydroxy platynecine	C10 H17 N O6	247.10559
21	propyl supinidine-type retronecine/heliotridine	C11 H17 N O2	195.12593
22	propyl retronecine/heliotridine	C11 H17 N O3	211.12084
23	propyl N-oxide/hydroxy retronecine/heliotridine	C11 H17 N O4	227.11576
24	propyl dihydroxy/N-oxide retronecine/heliotridine	C11 H17 N O5	243.11067
25	propyl trihydroxy/N-oxide retronecine/heliotridine	C11 H17 N O6	259.10559
26	propyl supinidine-type platynecine	C11 H19 N O2	197.14158
27	propyl platynecine	C11 H19 N O3	213.13649
28	propyl N-oxide/hydroxy platynecine	C11 H19 N O4	229.13141
29	propyl dihydroxy/N-oxide platynecine	C11 H19 N O5	245.12632
30	propyl trihydroxy/N-oxide platynecine	C11 H19 N O6	261.12124
31	butyryl supinidine-type retronecine/heliotridine	C12 H19 N O2	209.14158
32	butyryl retronecine/heliotridine	C12 H19 N O3	225.13649
33	butyryl N-oxide/hydroxy retronecine/heliotridine	C12 H19 N O4	241.13141
34	butyryl dihydroxy/N-oxide retronecine/heliotridine	C12 H19 N O5	257.12632
35	butyryl trihydroxy/N-oxide retronecine/heliotridine	C12 H19 N O6	273.12124
36	butyryl supinidine-type platynecine	C12 H21 N O2	211.15723
37	butyryl platynecine	C12 H21 N O3	227.15214
38	butyryl N-oxide/hydroxy platynecine	C12 H21 N O4	243.14706
39	butyryl dihydroxy/N-oxide platynecine	C12 H21 N O5	259.14197
40	butyryl trihydroxy/N-oxide platynecine	C12 H21 N O6	275.13689
41	angeloyl/tigloyl/senecioyl supinidine-type retronecine/heliotridine	C13 H19 N O2	221.14158
42	angeloyl/tigloyl/senecioyl retronecine/heliotridine	C13 H19 N O3	237.13649
43	angeloyl/tigloyl/senecioyl N-oxide/hydroxy retronecine/heliotridine	C13 H19 N O4	253.13141
44	angeloyl/tigloyl/senecioyl dihydroxy/N-oxide retronecine/heliotridine	C13 H19 N O5	269.12632
45	angeloyl/tigloyl/senecioyl trihydroxy/N-oxide retronecine/heliotridine	C13 H19 N O6	285.12124
46	angeloyl/tigloyl/senecioyl supinidine-type platynecine	C13 H21 N O2	223.15723
47	angeloyl/tigloyl/senecioyl platynecine	C13 H21 N O3	239.15214
48	angeloyl/tigloyl/senecioyl N-oxide/hydroxy platynecine	C13 H21 N O4	255.14706
49	angeloyl/tigloyl/senecioyl dihydroxy/N-oxide platynecine	C13 H21 N O5	271.14197
50	angeloyl/tigloyl/senecioyl trihydroxy/N-oxide platynecine	C13 H21 N O6	287.13689
51	methylbutyryl supinidine-type retronecine/heliotridine	C13 H21 N O2	223.15723
52	methylbutyryl retronecine/heliotridine	C13 H21 N O3	239.15214
53	methylbutyryl N-oxide/hydroxy retronecine/heliotridine	C13 H21 N O4	255.14706
54	methylbutyryl dihydroxy/N-oxide retronecine/heliotridine	C13 H21 N O5	271.14197
55	methylbutyryl trihydroxy/N-oxide retronecine/heliotridine	C13 H21 N O6	287.13689
56	methylbutyryl supinidine-type platynecine	C13 H23 N O2	225.17288
57	methylbutyryl platynecine	C13 H23 N O3	241.16779
58	methylbutyryl N-oxide/hydroxy platynecine	C13 H23 N O4	257.16271
59	methylbutyryl dihydroxy/N-oxide platynecine	C13 H23 N O5	273.15762
60	methylbutyryl trihydroxy/N-oxide platynecine	C13 H23 N O6	289.15254

ID	Name	Formula	Molecular Weight (Da)
61	dimethylbutyryl supinidine-type retronecine/heliotridine	C14 H23 N O2	237.17288
62	dimethylbutyryl retronecine/heliotridine	C14 H23 N O3	253.16779
63	dimethylbutyryl N-oxide/hydroxy retronecine/heliotridine	C14 H23 N O4	269.16271
64	dimethylbutyryl dihydroxy/N-oxide retronecine/heliotridine	C14 H23 N O5	285.15762
65	dimethylbutyryl trihydroxy/N-oxide retronecine/heliotridine	C14 H23 N O6	301.15254
66	dimethylbutyryl supinidine-type platynecine	C14 H25 N O2	239.18853
67	dimethylbutyryl platynecine	C14 H25 N O3	255.18344
68	dimethylbutyryl N-oxide/hydroxy platynecine	C14 H25 N O4	271.17836
69	dimethylbutyryl dihydroxy/N-oxide platynecine	C14 H25 N O5	287.17327
70	dimethylbutyryl trihydroxy/N-oxide platynecine	C14 H25 N O6	303.16819
71	acetyl+angeloyl/tigloyl/senecioyl supinidine-type retronecine/heliotridine	C15 H21 N O3	263.15214
72	acetyl+angeloyl/tigloyl/senecioyl retronecine/heliotridine	C15 H21 N O4	279.14706
73	acetyl+angeloyl/tigloyl/senecioyl N-oxide/hydroxy retronecine/heliotridine	C15 H21 N O5	295.14197
74	acetyl+angeloyl/tigloyl/senecioyl dihydroxy/N-oxide retronecine/heliotridine	C15 H21 N O6	311.13689
75	acetyl+angeloyl/tigloyl/senecioyl trihydroxy/N-oxide retronecine/heliotridine	C15 H21 N O7	327.13180
76	latifolyl/hackelyl supinidine-type retronecine/heliotridine	C15 H21 N O5	295.14197
77	latifolyl/hackelyl retronecine/heliotridine	C15 H21 N O6	311.13689
78	latifolyl/hackelyl N-oxide/hydroxy retronecine/heliotridine	C15 H21 N O7	327.13180
79	latifolyl/hackelyl dihydroxy/N-oxide retronecine/heliotridine	C15 H21 N O8	343.12672
80	latifolyl/hackelyl trihydroxy/N-oxide retronecine/heliotridine	C15 H21 N O9	359.12163
81	acetyl+angeloyl/tigloyl/senecioyl supinidine-type platynecine	C15 H23 N O3	265.16779
82	acetyl+angeloyl/tigloyl/senecioyl platynecine	C15 H23 N O4	281.16271
83	acetyl+angeloyl/tigloyl/senecioyl N-oxide/hydroxy platynecine	C15 H23 N O5	297.15762
84	acetyl+angeloyl/tigloyl/senecioyl dihydroxy/N-oxide platynecine	C15 H23 N O6	313.15254
85	acetyl+angeloyl/tigloyl/senecioyl trihydroxy/N-oxide platynecine	C15 H23 N O7	329.14745
86	acetyl+methylbutyryl supinidine-type retronecine/heliotridine	C15 H23 N O3	265.16779
87	acetyl+methylbutyryl retronecine/heliotridine	C15 H23 N O4	281.16271
88	acetyl+methylbutyryl N-oxide/hydroxy retronecine/heliotridine	C15 H23 N O5	297.15762
89	acetyl+methylbutyryl dihydroxy/N-oxide retronecine/heliotridine	C15 H23 N O6	313.15254
90	acetyl+methylbutyryl trihydroxy/N-oxide retronecine/heliotridine	C15 H23 N O7	329.14745
91	latifolyl/hackelyl supinidine-type platynecine	C15 H23 N O5	297.15762
92	latifolyl/hackelyl platynecine	C15 H23 N O6	313.15254
93	latifolyl/hackelyl N-oxide/hydroxy platynecine	C15 H23 N O7	329.14745
94	latifolyl/hackelyl dihydroxy/N-oxide platynecine	C15 H23 N O8	345.14237
95	latifolyl/hackelyl trihydroxy/N-oxide platynecine	C15 H23 N O9	361.13728
96	trachelantyl/viridifloryl supinidine-type retronecine/heliotridine	C15 H25 N O4	283.17836
97	trachelantyl/viridifloryl retronecine/heliotridine	C15 H25 N O5	299.17327
98	trachelantyl/viridifloryl N-oxide/hydroxy retronecine/heliotridine	C15 H25 N O6	315.16819
99	trachelantyl/viridifloryl dihydroxy/N-oxide retronecine/heliotridine	C15 H25 N O7	331.16310
100	trachelantyl/viridifloryl trihydroxy/N-oxide retronecine/heliotridine	C15 H25 N O8	347.15802
101	acetyl+methylbutyryl supinidine-type platynecine	C15 H25 N O3	267.18344
102	acetyl+methylbutyryl platynecine	C15 H25 N O4	283.17836
103	acetyl+methylbutyryl N-oxide/hydroxy platynecine	C15 H25 N O5	299.17327
104	acetyl+methylbutyryl dihydroxy/N-oxide platynecine	C15 H25 N O6	315.16819
105	acetyl+methylbutyryl trihydroxy/N-oxide platynecine	C15 H25 N O7	331.16310
106	trachelantyl/viridifloryl supinidine-type platynecine	C15 H27 N O4	285.19401
107	trachelantyl/viridifloryl platynecine	C15 H27 N O5	301.18892
108	trachelantyl/viridifloryl N-oxide/hydroxy platynecine	C15 H27 N O6	317.18384
109	trachelantyl/viridifloryl dihydroxy/N-oxide platynecine	C15 H27 N O7	333.17875
110	trachelantyl/viridifloryl trihydroxy/N-oxide platynecine	C15 H27 N O8	349.17367
111	heliotridyl/curassavoyl supinidine-type retronecine/heliotridine	C16 H27 N O4	297.19401
112	heliotridyl/curassavoyl retronecine/heliotridine	C16 H27 N O5	313.18892
113	heliotridyl/curassavoyl N-oxide/hydroxy retronecine/heliotridine	C16 H27 N O6	329.18384
114	heliotridyl/curassavoyl dihydroxy/N-oxide retronecine/heliotridine	C16 H27 N O7	345.17875
115	heliotridyl/curassavoyl trihydroxy/N-oxide retronecine/heliotridine	C16 H27 N O8	361.17367
116	heliotridyl/curassavoyl supinidine-type platynecine	C16 H29 N O4	299.20966
117	heliotridyl/curassavoyl platynecine	C16 H29 N O5	315.20457
118	heliotridyl/curassavoyl N-oxide/hydroxy platynecine	C16 H29 N O6	331.19949
119	heliotridyl/curassavoyl dihydroxy/N-oxide platynecine	C16 H29 N O7	347.19440
120	heliotridyl/curassavoyl trihydroxy/N-oxide platynecine	C16 H29 N O8	363.18932
121	acetyl+latifolyl/hackelyl supinidine-type retronecine/heliotridine	C17 H23 N O6	337.15254
122	acetyl+latifolyl/hackelyl retronecine/heliotridine	C17 H23 N O7	353.14745

ID	Name	Formula	Molecular Weight (Da)
123	acetyl+latifolyl/hackelyl N-oxide/hydroxy retronecine/heliotridine	C17 H23 N O8	369.14237
124	acetyl+latifolyl/hackelyl dihydroxy/N-oxide retronecine/heliotridine	C17 H23 N O9	385.13728
125	acetyl+latifolyl/hackelyl trihydroxy/N-oxide retronecine/heliotridine	C17 H23 N O10	401.13220
126	acetyl+latifolyl/hackelyl supinidine-type platynecine	C17 H25 N O6	339.16819
127	acetyl+latifolyl/hackelyl platynecine	C17 H25 N O7	355.16310
128	acetyl+latifolyl/hackelyl N-oxide/hydroxy platynecine	C17 H25 N O8	371.15802
129	acetyl+latifolyl/hackelyl dihydroxy/N-oxide platynecine	C17 H25 N O9	387.15293
130	acetyl+latifolyl/hackelyl trihydroxy/N-oxide platynecine	C17 H25 N O10	403.14785
131	acetyl+trachelantyl/viriflоryl supinidine-type retronecine/heliotridine	C17 H27 N O5	325.18892
132	acetyl+trachelantyl/viriflоryl retronecine/heliotridine	C17 H27 N O6	341.18384
133	acetyl+trachelantyl/viriflоryl N-oxide/hydroxy retronecine/heliotridine	C17 H27 N O7	357.17875
134	acetyl+trachelantyl/viriflоryl dihydroxy/N-oxide retronecine/heliotridine	C17 H27 N O8	373.17367
135	acetyl+trachelantyl/viriflоryl trihydroxy/N-oxide retronecine/heliotridine	C17 H27 N O9	389.16858
136	acetyl+trachelantyl/viriflоryl supinidine-type platynecine	C17 H29 N O5	327.20457
137	acetyl+trachelantyl/viriflоryl platynecine	C17 H29 N O6	343.19949
138	acetyl+trachelantyl/viriflоryl N-oxide/hydroxy platynecine	C17 H29 N O7	359.19440
139	acetyl+trachelantyl/viriflоryl dihydroxy/N-oxide platynecine	C17 H29 N O8	375.18932
140	acetyl+trachelantyl/viriflоryl trihydroxy/N-oxide platynecine	C17 H29 N O9	391.18423
141	diangeloyl/tigloyl/senecioyl supinidine-type retronecine/heliotridine	C18 H25 N O3	303.18344
142	diangeloyl/tigloyl/senecioyl retronecine/heliotridine	C18 H25 N O4	319.17836
143	diangeloyl/tigloyl/senecioyl N-oxide/hydroxy retronecine/heliotridine	C18 H25 N O5	335.17327
144	diangeloyl/tigloyl/senecioyl dihydroxy/N-oxide retronecine/heliotridine	C18 H25 N O6	351.16819
145	diangeloyl/tigloyl/senecioyl trihydroxy/N-oxide retronecine/heliotridine	C18 H25 N O7	367.16310
146	diangeloyl/tigloyl/senecioyl supinidine-type platynecine	C18 H27 N O3	305.19909
147	diangeloyl/tigloyl/senecioyl platynecine	C18 H27 N O4	321.19401
148	diangeloyl/tigloyl/senecioyl N-oxide/hydroxy platynecine	C18 H27 N O5	337.18892
149	diangeloyl/tigloyl/senecioyl dihydroxy/N-oxide platynecine	C18 H27 N O6	353.18384
150	diangeloyl/tigloyl/senecioyl trihydroxy/N-oxide platynecine	C18 H27 N O7	369.17875
151	methylbutyryl+angeloyl/tigloyl/senecioyl supinidine-type retronecine/heliotridine	C18 H27 N O3	305.19909
152	methylbutyryl+angeloyl/tigloyl/senecioyl retronecine/heliotridine	C18 H27 N O4	321.19401
153	methylbutyryl+angeloyl/tigloyl/senecioyl N-oxide/hydroxy retronecine/heliotridine	C18 H27 N O5	337.18892
154	methylbutyryl+angeloyl/tigloyl/senecioyl dihydroxy/N-oxide retronecine/heliotridine	C18 H27 N O6	353.18384
155	methylbutyryl+angeloyl/tigloyl/senecioyl trihydroxy/N-oxide retronecine/heliotridine	C18 H27 N O7	369.17875
156	acetyl+heliotridyl/curassavoyl supinidine-type retronecine/heliotridine	C18 H29 N O5	339.20457
157	acetyl+heliotridyl/curassavoyl retronecine/heliotridine	C18 H29 N O6	355.19949
158	acetyl+heliotridyl/curassavoyl+N-oxide/hydroxy retronecine/heliotridine	C18 H29 N O7	371.19440
159	acetyl+heliotridyl/curassavoyl+dihydroxy/N-oxide retronecine/heliotridine	C18 H29 N O8	387.18932
160	acetyl+heliotridyl/curassavoyl+trihydroxy/N-oxide retronecine/heliotridine	C18 H29 N O9	403.18423
161	methylbutyryl+angeloyl/tigloyl/senecioyl supinidine-type platynecine	C18 H29 N O3	307.21474
162	methylbutyryl+angeloyl/tigloyl/senecioyl platynecine	C18 H29 N O4	323.20966
163	methylbutyryl+angeloyl/tigloyl/senecioyl N-oxide/hydroxy platynecine	C18 H29 N O5	339.20457
164	methylbutyryl+angeloyl/tigloyl/senecioyl dihydroxy/N-oxide platynecine	C18 H29 N O6	355.19949
165	methylbutyryl+angeloyl/tigloyl/senecioyl trihydroxy/N-oxide platynecine	C18 H29 N O7	371.19440
166	acetyl+heliotridyl/curassavoyl supinidine-type platynecine	C18 H31 N O5	341.22022
167	acetyl+heliotridyl/curassavoyl platynecine	C18 H31 N O6	357.21514
168	acetyl+heliotridyl/curassavoyl+N-oxide/hydroxy platynecine	C18 H31 N O7	373.21005
169	acetyl+heliotridyl/curassavoyl+dihydroxy/N-oxide platynecine	C18 H31 N O8	389.20497
170	acetyl+heliotridyl/curassavoyl+trihydroxy/N-oxide platynecine	C18 H31 N O9	405.19988
171	acetyl+diangeloyl/tigloyl/senecioyl supinidine-type retronecine/heliotridine	C20 H27 N O4	345.19401
172	acetyl+diangeloyl/tigloyl/senecioyl retronecine/heliotridine	C20 H27 N O5	361.18892
173	acetyl+diangeloyl/tigloyl/senecioyl N-oxide/hydroxy retronecine/heliotridine	C20 H27 N O6	377.18384
174	acetyl+diangeloyl/tigloyl/senecioyl dihydroxy/N-oxide retronecine/heliotridine	C20 H27 N O7	393.17875
175	acetyl+diangeloyl/tigloyl/senecioyl trihydroxy/N-oxide retronecine/heliotridine	C20 H27 N O8	409.17367
176	angeloyl/tigloyl/senecioyl+latifolyl/hackelyl supinidine-type retronecine/heliotridine	C20 H27 N O6	377.18384
177	angeloyl/tigloyl/senecioyl+latifolyl/hackelyl retronecine/heliotridine	C20 H27 N O7	393.17875
178	angeloyl/tigloyl/senecioyl+latifolyl/hackelyl N-oxide/hydroxy retronecine/heliotridine	C20 H27 N O8	409.17367
179	angeloyl/tigloyl/senecioyl+latifolyl/hackelyl dihydroxy/N-oxide retronecine/heliotridine	C20 H27 N O9	425.16858
180	angeloyl/tigloyl/senecioyl+latifolyl/hackelyl trihydroxy/N-oxide retronecine/heliotridine	C20 H27 N O10	441.1635
181	acetyl+diangeloyl/tigloyl/senecioyl supinidine-type platynecine	C20 H29 N O4	347.20966
182	acetyl+diangeloyl/tigloyl/senecioyl platynecine	C20 H29 N O5	363.20457
183	acetyl+diangeloyl/tigloyl/senecioyl N-oxide/hydroxy platynecine	C20 H29 N O6	379.19949
184	acetyl+diangeloyl/tigloyl/senecioyl dihydroxy/N-oxide platynecine	C20 H29 N O7	395.19440

ID	Name	Formula	Molecular Weight (Da)
185	acetyl+diangeloyl/tigloyl/senecioyl trihydroxy/N-oxide platynecine	C20 H29 N O8	411.18932
186	acetyl+methylbutyryl+angeloyl/tigloyl/senecioyl retronecine/heliotridine	C20 H29 N O4	347.20966
187	acetyl+methylbutyryl+angeloyl/tigloyl/senecioyl retronecine/heliotridine	C20 H29 N O5	363.20457
188	acetyl+methylbutyryl+angeloyl/tigloyl/senecioyl N-oxide/hydroxy retronecine/heliotridine	C20 H29 N O6	379.19949
189	acetyl+methylbutyryl+angeloyl/tigloyl/senecioyl dihydroxy/N-oxide retronecine/heliotridine	C20 H29 N O7	395.19440
190	acetyl+methylbutyryl+angeloyl/tigloyl/senecioyl trihydroxy/N-oxide retronecine/heliotridine	C20 H29 N O8	411.18932
191	angeloyl/tigloyl/senecioyl+latifolyl/hackelyl supinidine-type platynecine	C20 H29 N O6	379.19949
192	angeloyl/tigloyl/senecioyl+latifolyl/hackelyl platynecine	C20 H29 N O7	395.19440
193	angeloyl/tigloyl/senecioyl+latifolyl/hackelyl N-oxide/hydroxy platynecine	C20 H29 N O8	411.18932
194	angeloyl/tigloyl/senecioyl+latifolyl/hackelyl dihydroxy/N-oxide platynecine	C20 H29 N O9	427.18423
195	angeloyl/tigloyl/senecioyl+latifolyl/hackelyl trihydroxy/N-oxide platynecine	C20 H29 N O10	443.17915
196	angeloyl/tigloyl/senecioyl+trachelantyl/viridifloryl retronecine/heliotridine	C20 H31 N O5	365.22022
197	angeloyl/tigloyl/senecioyl+trachelantyl/viridifloryl retronecine/heliotridine	C20 H31 N O6	381.21514
198	angeloyl/tigloyl/senecioyl+trachelantyl/viridifloryl N-oxide/hydroxy retronecine/heliotridine	C20 H31 N O7	397.21005
199	angeloyl/tigloyl/senecioyl+trachelantyl/viridifloryl dihydroxy/N-oxide retronecine/heliotridine	C20 H31 N O8	413.20497
200	angeloyl/tigloyl/senecioyl+trachelantyl/viridifloryl trihydroxy/N-oxide retronecine/heliotridine	C20 H31 N O9	429.19988
201	acetyl+methylbutyryl+angeloyl/tigloyl/senecioyl platynecine	C20 H31 N O4	349.22531
202	acetyl+methylbutyryl+angeloyl/tigloyl/senecioyl platynecine	C20 H31 N O5	365.22022
203	acetyl+methylbutyryl+angeloyl/tigloyl/senecioyl N-oxide/hydroxy platynecine	C20 H31 N O6	381.21514
204	acetyl+methylbutyryl+angeloyl/tigloyl/senecioyl dihydroxy/N-oxide platynecine	C20 H31 N O7	397.21005
205	acetyl+methylbutyryl+angeloyl/tigloyl/senecioyl trihydroxy/N-oxide platynecine	C20 H31 N O8	413.20497
206	angeloyl/tigloyl/senecioyl+trachelantyl/viridifloryl platynecine	C20 H33 N O5	367.23587
207	angeloyl/tigloyl/senecioyl+trachelantyl/viridifloryl platynecine	C20 H33 N O6	383.23079
208	angeloyl/tigloyl/senecioyl+trachelantyl/viridifloryl N-oxide/hydroxy platynecine	C20 H33 N O7	399.22570
209	angeloyl/tigloyl/senecioyl+trachelantyl/viridifloryl dihydroxy/N-oxide platynecine	C20 H33 N O8	415.22062
210	angeloyl/tigloyl/senecioyl+trachelantyl/viridifloryl trihydroxy/N-oxide platynecine	C20 H33 N O9	431.21553
211	methylbutyryl+trachelantyl/viridifloryl retronecine/heliotridine	C20 H33 N O5	367.23587
212	methylbutyryl+trachelantyl/viridifloryl retronecine/heliotridine	C20 H33 N O6	383.23079
213	methylbutyryl+trachelantyl/viridifloryl N-oxide/hydroxy retronecine/heliotridine	C20 H33 N O7	399.22570
214	methylbutyryl+trachelantyl/viridifloryl dihydroxy/N-oxide retronecine/heliotridine	C20 H33 N O8	415.22062
215	methylbutyryl+trachelantyl/viridifloryl trihydroxy/N-oxide retronecine/heliotridine	C20 H33 N O9	431.21553
216	methylbutyryl+trachelantyl/viridifloryl platynecine	C20 H35 N O5	369.25152
217	methylbutyryl+trachelantyl/viridifloryl platynecine	C20 H35 N O6	385.24644
218	methylbutyryl+trachelantyl/viridifloryl N-oxide/hydroxy platynecine	C20 H35 N O7	401.24135
219	methylbutyryl+trachelantyl/viridifloryl dihydroxy/N-oxide platynecine	C20 H35 N O8	417.23627
220	methylbutyryl+trachelantyl/viridifloryl trihydroxy/N-oxide platynecine	C20 H35 N O9	433.23118
221	angeloyl/tigloyl/senecioyl+heliotridyl/curassavoyl supinidine-type retronecine/heliotridine	C21 H33 N O5	379.23587
222	angeloyl/tigloyl/senecioyl+heliotridyl/curassavoyl retronecine/heliotridine	C21 H33 N O6	395.23079
223	angeloyl/tigloyl/senecioyl+heliotridyl/curassavoyl N-oxide/hydroxy retronecine/heliotridine	C21 H33 N O7	411.22570
224	angeloyl/tigloyl/senecioyl+heliotridyl/curassavoyl dihydroxy/N-oxide retronecine/heliotridine	C21 H33 N O8	427.22062
225	angeloyl/tigloyl/senecioyl+heliotridyl/curassavoyl trihydroxy/N-oxide retronecine/heliotridine	C21 H33 N O9	443.21553
226	angeloyl/tigloyl/senecioyl+heliotridyl/curassavoyl supinidine-type platynecine	C21 H35 N O5	381.25152
227	angeloyl/tigloyl/senecioyl+heliotridyl/curassavoyl platynecine	C21 H35 N O6	397.24644
228	angeloyl/tigloyl/senecioyl+heliotridyl/curassavoyl N-oxide/hydroxy platynecine	C21 H35 N O7	413.24135
229	angeloyl/tigloyl/senecioyl+heliotridyl/curassavoyl dihydroxy/N-oxide platynecine	C21 H35 N O8	429.23627
230	angeloyl/tigloyl/senecioyl+heliotridyl/curassavoyl trihydroxy/N-oxide platynecine	C21 H35 N O9	445.23118
231	methylbutyryl+heliotridyl/curassavoyl supinidine-type retronecine/heliotridine	C21 H35 N O5	381.25152
232	methylbutyryl+heliotridyl/curassavoyl retronecine/heliotridine	C21 H35 N O6	397.24644
233	methylbutyryl+heliotridyl/curassavoyl N-oxide/hydroxy retronecine/heliotridine	C21 H35 N O7	413.24135
234	methylbutyryl+heliotridyl/curassavoyl dihydroxy/N-oxide retronecine/heliotridine	C21 H35 N O8	429.23627
235	methylbutyryl+heliotridyl/curassavoyl trihydroxy/N-oxide retronecine/heliotridine	C21 H35 N O9	445.23118
236	methylbutyryl+heliotridyl/curassavoyl supinidine-type platynecine	C21 H37 N O5	383.26717
237	methylbutyryl+heliotridyl/curassavoyl platynecine	C21 H37 N O6	399.26209
238	methylbutyryl+heliotridyl/curassavoyl N-oxide/hydroxy platynecine	C21 H37 N O7	415.25700
239	methylbutyryl+heliotridyl/curassavoyl dihydroxy/N-oxide platynecine	C21 H37 N O8	431.25192
240	methylbutyryl+heliotridyl/curassavoyl trihydroxy/N-oxide platynecine	C21 H37 N O9	447.24683
241	acetyl+angeloyl/tigloyl/senecioyl+latifolyl/hackelyl retronecine/heliotridine	C22 H29 N O8	435.18932
242	acetyl+angeloyl/tigloyl/senecioyl+latifolyl/hackelyl N-oxide/hydroxy retronecine/heliotridine	C22 H29 N O9	451.18423
243	acetyl+angeloyl/tigloyl/senecioyl+latifolyl/hackelyl dihydroxy/N-oxide retronecine/heliotridine	C22 H29 N O10	467.17915
244	acetyl+angeloyl/tigloyl/senecioyl+latifolyl/hackelyl trihydroxy/N-oxide retronecine/heliotridine	C22 H29 N O11	483.17406
245	acetyl+angeloyl/tigloyl/senecioyl+latifolyl/hackelyl platynecine	C22 H31 N O8	437.20497
246	acetyl+angeloyl/tigloyl/senecioyl+latifolyl/hackelyl N-oxide/hydroxy platynecine	C22 H31 N O9	453.19988

ID	Name	Formula	Molecular Weight (Da)
247	acetyl+angeloyl/tigloyl/senecioyl+latifolyl/hackelyl dihydroxy/N-oxide platynecine	C22 H31 N O10	469.19480
248	acetyl+angeloyl/tigloyl/senecioyl+latifolyl/hackelyl trihydroxy/N-oxide platynecine	C22 H31 N O11	485.18971
249	acetyl+angeloyl/tigloyl/senecioyl+trachelantyl/viridifloryl retronecine/heliotridine	C22 H33 N O7	423.22570
250	acetyl+angeloyl/tigloyl/senecioyl+trachelantyl/viridifloryl N-oxide/hydroxy retronecine/heliotridine	C22 H33 N O8	439.22062
251	acetyl+angeloyl/tigloyl/senecioyl+trachelantyl/viridifloryl dihydroxy/N-oxide retronecine/heliotridine	C22 H33 N O9	455.21553
252	acetyl+angeloyl/tigloyl/senecioyl+trachelantyl/viridifloryl trihydroxy/N-oxide retronecine/heliotridine	C22 H33 N O10	471.21045
253	acetyl+angeloyl/tigloyl/senecioyl+trachelantyl/viridifloryl platynecine	C22 H35 N O7	425.24135
254	acetyl+angeloyl/tigloyl/senecioyl+trachelantyl/viridifloryl N-oxide/hydroxy platynecine	C22 H35 N O8	441.23627
255	acetyl+angeloyl/tigloyl/senecioyl+trachelantyl/viridifloryl dihydroxy/N-oxide platynecine	C22 H35 N O9	457.23118
256	acetyl+angeloyl/tigloyl/senecioyl+trachelantyl/viridifloryl trihydroxy/N-oxide platynecine	C22 H35 N O10	473.22610
257	acetyl+methylbutyryl+trachelantyl/viridifloryl retronecine/heliotridine	C22 H35 N O7	425.24135
258	acetyl+methylbutyryl+trachelantyl/viridifloryl N-oxide/hydroxy retronecine/heliotridine	C22 H35 N O8	441.23627
259	acetyl+methylbutyryl+trachelantyl/viridifloryl dihydroxy/N-oxide retronecine/heliotridine	C22 H35 N O9	457.23118
260	acetyl+methylbutyryl+trachelantyl/viridifloryl trihydroxy/N-oxide retronecine/heliotridine	C22 H35 N O10	473.22610
261	ditrachelantyl/viridifloryl retronecine/heliotridine	C22 H37 N O8	443.25192
262	ditrachelantyl/viridifloryl N-oxide/hydroxy retronecine/heliotridine	C22 H37 N O9	459.24683
263	ditrachelantyl/viridifloryl dihydroxy/N-oxide retronecine/heliotridine	C22 H37 N O10	475.24175
264	ditrachelantyl/viridifloryl trihydroxy/N-oxide retronecine/heliotridine	C22 H37 N O11	491.23666
265	acetyl+methylbutyryl+trachelantyl/viridifloryl platynecine	C22 H37 N O7	427.25700
266	acetyl+methylbutyryl+trachelantyl/viridifloryl N-oxide/hydroxy platynecine	C22 H37 N O8	443.25192
267	acetyl+methylbutyryl+trachelantyl/viridifloryl dihydroxy/N-oxide platynecine	C22 H37 N O9	459.24683
268	acetyl+methylbutyryl+trachelantyl/viridifloryl trihydroxy/N-oxide platynecine	C22 H37 N O10	475.24175
269	ditrachelantyl/viridifloryl platynecine	C22 H39 N O8	445.26757
270	ditrachelantyl/viridifloryl N-oxide/hydroxy platynecine	C22 H39 N O9	461.26248
271	ditrachelantyl/viridifloryl dihydroxy/N-oxide platynecine	C22 H39 N O10	477.25740
272	ditrachelantyl/viridifloryl trihydroxy/N-oxide platynecine	C22 H39 N O11	493.25231
273	acetyl+angeloyl/tigloyl/senecioyl+heliotridyl/curassavoyl retronecine/heliotridine	C23 H35 N O7	437.24135
274	acetyl+angeloyl/tigloyl/senecioyl+heliotridyl/curassavoyl N-oxide/hydroxy retronecine/heliotridine	C23 H35 N O8	453.23627
275	acetyl+angeloyl/tigloyl/senecioyl+heliotridyl/curassavoyl dihydroxy/N-oxide retronecine/heliotridine	C23 H35 N O9	469.23118
276	acetyl+angeloyl/tigloyl/senecioyl+heliotridyl/curassavoyl trihydroxy/N-oxide retronecine/heliotridine	C23 H35 N O10	485.22610
277	acetyl+angeloyl/tigloyl/senecioyl+heliotridyl/curassavoyl platynecine	C23 H37 N O7	439.25700
278	acetyl+angeloyl/tigloyl/senecioyl+heliotridyl/curassavoyl N-oxide/hydroxy platynecine	C23 H37 N O8	455.25192
279	acetyl+angeloyl/tigloyl/senecioyl+heliotridyl/curassavoyl dihydroxy/N-oxide platynecine	C23 H37 N O9	471.24683
280	acetyl+angeloyl/tigloyl/senecioyl+heliotridyl/curassavoyl trihydroxy/N-oxide platynecine	C23 H37 N O10	487.24175
281	diangeloyl/tigloyl/senecioyl+heliotridyl/curassavoyl retronecine/heliotridine	C26 H39 N O7	477.27265
282	diangeloyl/tigloyl/senecioyl+heliotridyl/curassavoyl N-oxide/hydroxy retronecine/heliotridine	C26 H39 N O8	493.26757
283	diangeloyl/tigloyl/senecioyl+heliotridyl/curassavoyl dihydroxy/N-oxide retronecine/heliotridine	C26 H39 N O9	509.26248
284	diangeloyl/tigloyl/senecioyl+heliotridyl/curassavoyl trihydroxy/N-oxide retronecine/heliotridine	C26 H39 N O10	525.25740
285	diangeloyl/tigloyl/senecioyl+heliotridyl/curassavoyl platynecine	C26 H41 N O7	479.28830
286	diangeloyl/tigloyl/senecioyl+heliotridyl/curassavoyl N-oxide/hydroxy platynecine	C26 H41 N O8	495.28322
287	diangeloyl/tigloyl/senecioyl+heliotridyl/curassavoyl dihydroxy/N-oxide platynecine	C26 H41 N O9	511.27813
288	diangeloyl/tigloyl/senecioyl+heliotridyl/curassavoyl trihydroxy/N-oxide platynecine	C26 H41 N O10	527.27305
289	acetyl+diangeloyl/tigloyl/senecioyl+heliotridyl/curassavoyl retronecine/heliotridine	C28 H41 N O8	519.28322
290	acetyl+diangeloyl/tigloyl/senecioyl+heliotridyl/curassavoyl N-oxide/hydroxy retronecine/heliotridine	C28 H41 N O9	535.27813
291	acetyl+diangeloyl/tigloyl/senecioyl+heliotridyl/curassavoyl dihydroxy/N-oxide retronecine/heliotridine	C28 H41 N O10	551.27305
292	acetyl+diangeloyl/tigloyl/senecioyl+heliotridyl/curassavoyl trihydroxy/N-oxide retronecine/heliotridine	C28 H41 N O11	567.26796
293	acetyl+diangeloyl/tigloyl/senecioyl+heliotridyl/curassavoyl platynecine	C28 H43 N O8	521.29887
294	acetyl+diangeloyl/tigloyl/senecioyl+heliotridyl/curassavoyl N-oxide/hydroxy platynecine	C28 H43 N O9	537.29378
295	acetyl+diangeloyl/tigloyl/senecioyl+heliotridyl/curassavoyl dihydroxy/N-oxide platynecine	C28 H43 N O10	553.28870
296	acetyl+diangeloyl/tigloyl/senecioyl+heliotridyl/curassavoyl trihydroxy/N-oxide platynecine	C28 H43 N O11	569.28361
