# Supplementary material for: Bioassay-directed analysis-based identification of relevant pyrrolizidine alkaloids
Source: Arch Toxicol. 2022 May 24;96(8):2299–317. doi: 10.1007/s00204-022-03308-z (PMC9217854; doi:10.1007/s00204-022-03308-z)
Supplement: Supplementary file 8 — Supplementary file8 (PDF 111 KB) [file 204_2022_3308_MOESM8_ESM.pdf]

Supplementary Table 5. Concentrations of PAs used in the equipotent ternary mixture experiment.

A. Concentrations for testing single compounds

**Europine (µM)**	**Heliotrine (µM)**	**Lasiocarpine (µM)**
12.5	12.5	0.78125
25	25	15.625
50	50	3.125
100	100	6.25
200	200	12.5
400	400	25

B. Concentrations for ternary mixtures

**RPF**	**1**	**1.23**	**16.33**	
**Compound**	**Europine (µM)**	**Heliotrine (µM)**	**Lasiocarpine (µM)**	**Europine equivalent (µM)**
Mixture 1	2.08	1.69	0.13	6.25
Mixture 2	4.17	3.39	0.26	12.5
Mixture 3	8.33	6.78	0.51	25
Mixture 4	16.67	13.55	1.02	50
Mixture 5	33.33	27.10	2.04	100
Mixture 6	66.67	54.20	4.08	200
Mixture 7	133.33	108.40	8.16	400
