# Supplementary material for: Bioassay-directed analysis-based identification of relevant pyrrolizidine alkaloids
Source: Arch Toxicol. 2022 May 24;96(8):2299–317. doi: 10.1007/s00204-022-03308-z (PMC9217854; doi:10.1007/s00204-022-03308-z)
Supplement: Supplementary file 9 — Supplementary file9 (PDF 422 KB) [file 204_2022_3308_MOESM9_ESM.pdf]

No	Ester type	(tentative) name	in-house PA list	Formula	Annot. DeltaMass [ppm]	Calc. MW	RT [min]	Assigned annotation level	# ChemSpider Results	# mzCloud Results	mzCloud Best Match	mzCloud Best Match Confidence	Mass List Match: Open Chain PAs final Mass list with dimers
1	ME	angeloyl heliotridine (or isomer)	angeloyl/tigloyl/senecioyl retronecine/heliotridine	C13 H19 N O3	0.28	237.1366	8.96	3	259	0			Single match found
2	ME	angeloyl heliotridine (or isomer)	angeloyl/tigloyl/senecioyl retronecine/heliotridine	C13 H19 N O3	-0.51	237.1364	9.18	3	259	0			Single match found
3	ME	angeloyl heliotridine (or isomer)	angeloyl/tigloyl/senecioyl retronecine/heliotridine	C13 H19 N O3	-1.10	237.1362	11.52	3	259	0			Single match found
4	ME	angeloyl heliotridine N-oxide (or isomer)	angeloyl/tigloyl/senecioyl N-oxide/hydroxy retronecine/heliotridine	C13 H19 N O4	-0.03	253.1314	5.29	3	136	4	77	8.9	Single match found
5	ME	angeloyl heliotridine N-oxide (or isomer)	angeloyl/tigloyl/senecioyl N-oxide/hydroxy retronecine/heliotridine	C13 H19 N O4	-1.02	253.1312	6.59	3	136	0			Single match found
6	ME	angeloyl heliotridine N-oxide (or isomer)	angeloyl/tigloyl/senecioyl N-oxide/hydroxy retronecine/heliotridine	C13 H19 N O4	-1.68	253.1310	6.73	3	136	0			Single match found
7	ME	hydroxyangeloyl heliotridine (or isomer)	angeloyl/tigloyl/senecioyl N-oxide/hydroxy retronecine/heliotridine	C13 H19 N O4	-1.47	253.1310	8.59	3	136	0			Single match found
8	ME	hydroxyangeloyl heliotridine N-oxide (or isome	angeloyl/tigloyl/senecioyl dihydroxy retronecine/heliotridine	C13 H19 N O5	-1.08	269.1260	4.02	3	30	0			Single match found
9	ME	dihydroxyangeloyl heliotridine (or isomer)	angeloyl/tigloyl/senecioyl dihydroxy retronecine/heliotridine	C13 H19 N O5	-2.01	269.1258	6.34	3	30	0			Single match found
10	PE	hydroxyangeloyl platynecine N-oxide (or isome	angeloyl/tigloyl/senecioyl dihydroxy platynecine	C13 H21 N O5	-0.37	271.1419	5.06	3	41	0			Multiple matches found
11	PE	dihydroxyangeloyl platynecine (or isomer)	angeloyl/tigloyl/senecioyl dihydroxy platynecine	C13 H21 N O5	-1.97	271.1414	6.57	3	41	0			Multiple matches found
12	PE	dihydroxyangeloyl platynecine N-oxide (or isor	angeloyl/tigloyl/senecioyl trihydroxy platynecine	C13 H21 N O6	-1.07	287.1366	2.48	3	26	0			Multiple matches found
13	ME	supinine	trachelantyl/viridifloryl supinidine-type retronecine/heliotridine	C15 H25 N O4	-0.48	283.1782	7.42	2	58	0			Multiple matches found
14	ME	supinine N-oxide	trachelantyl/viridifloryl retronecine/heliotridine	C15 H25 N O5	-1.94	299.1727	5.37	2	20	0			Multiple matches found
15	ME	echinatine	trachelantyl/viridifloryl retronecine/heliotridine	C15 H25 N O5	-1.58	299.1728	6.82	1	20	2	74	46.5	Multiple matches found
16	ME	rinderine	trachelantyl/viridifloryl retronecine/heliotridine	C15 H25 N O5	-1.54	299.1728	6.99	1	20	2	67.6	43.8	Multiple matches found
17	ME	echinatine N-oxide	trachelantyl/viridifloryl N-oxide/hydroxy retronecine/heliotridine	C15 H25 N O6	-2.03	315.1676	4.18	1	18	1	81.2	9.1	Multiple matches found
18	ME	rinderine N-oxide	trachelantyl/viridifloryl N-oxide/hydroxy retronecine/heliotridine	C15 H25 N O6	-1.76	315.1676	4.32	1	18	2	80.8	9	Multiple matches found
19	ME	5'-hydroxyrinderine	trachelantyl/viridifloryl N-oxide/hydroxy retronecine/heliotridine	C15 H25 N O6	-1.87	315.1676	4.92	2	18	0			Multiple matches found
20	ME	5'-hydroxyrinderine N-oxide	trachelantyl/viridifloryl dihydroxy retronecine/heliotridine	C15 H25 N O7	-2.84	331.1622	2.56	2	6	3	89.6	9.5	Multiple matches found
21	PE	trachelanthamine	trachelantyl/viridifloryl supinidine-type platynecine	C15 H27 N O4	-1.36	285.1936	6.55	1	40	0			Single match found
22	PE	hydroxytrachelanthamidine (or isomer)	trachelantyl/viridifloryl platynecine	C15 H27 N O5	-0.55	301.1888	5.15	3	17	0			Single match found
23	PE	hydroxytrachelanthamidine (or isomer)	trachelantyl/viridifloryl platynecine	C15 H27 N O5	-0.90	301.1887	5.24	3	17	0			Single match found
24	PE	trachelanthamine N-oxide	trachelantyl/viridifloryl platynecine	C15 H27 N O5	-1.89	301.1884	5.51	1	17	0			Single match found
25	PE	hydroxytrachelanthamidine (or isomer)	trachelantyl/viridifloryl platynecine	C15 H27 N O5	-0.92	301.1887	5.84	3	17	0			Single match found
26	PE	hydroxytrachelanthamidine (or isomer)	trachelantyl/viridifloryl platynecine	C15 H27 N O5	-1.38	301.1885	6.10	3	17	0			Single match found
27	PE	hydroxytrachelanthamidine (or isomer)	trachelantyl/viridifloryl platynecine	C15 H27 N O5	-1.83	301.1884	6.23	3	17	0			Single match found
28	PE	hydroxytrachelanthamidine (or isomer)	trachelantyl/viridifloryl platynecine	C15 H27 N O5	-1.28	301.1885	6.32	3	17	0			Single match found
29	PE	hydroxytrachelanthamidine (or isomer)	trachelantyl/viridifloryl platynecine	C15 H27 N O5	-1.15	301.1886	6.45	3	17	0			Single match found
30	PE	hydroxytrachelanthamidine (or isomer)	trachelantyl/viridifloryl platynecine	C15 H27 N O5	-1.22	301.1886	6.75	3	17	0			Single match found
31	PE	hydroxytrachelanthamidine N-oxide (or isome	trachelantyl/viridifloryl N-oxide/hydroxy platynecine	C15 H27 N O6	-1.82	317.1833	4.25	3	7	2	98.5	84.6	Single match found
32	PE	dihydroxytrachelanthamidine (or isomer)	trachelantyl/viridifloryl N-oxide/hydroxy platynecine	C15 H27 N O6	-1.76	317.1833	4.45	3	7	2	97.9	81.8	Single match found
33	PE	dihydroxytrachelanthamidine (or isomer)	trachelantyl/viridifloryl N-oxide/hydroxy platynecine	C15 H27 N O6	-1.41	317.1834	4.61	3	7	1	97.5	80.6	Single match found
34	PE	dihydroxytrachelanthamidine (or isomer)	trachelantyl/viridifloryl N-oxide/hydroxy platynecine	C15 H27 N O6	-1.11	317.1835	4.84	3	7	2	97.4	80.3	Single match found
35	PE	dihydroxytrachelanthamidine (or isomer)	trachelantyl/viridifloryl N-oxide/hydroxy platynecine	C15 H27 N O6	-1.35	317.1834	5.02	3	7	2	92.3	64.3	Single match found
36	PE	dihydroxytrachelanthamidine (or isomer)	trachelantyl/viridifloryl N-oxide/hydroxy platynecine	C15 H27 N O6	-0.90	317.1836	5.51	3	7	2	95.7	75.6	Single match found
37	PE	trihydroxytrachelanthamidine (or isomer)	trachelantyl/viridifloryl dihydroxy platynecine	C15 H27 N O7	-1.06	333.1784	3.76	3	5	4	73.5	46.2	Single match found
38	PE	trihydroxytrachelanthamidine (or isomer)	trachelantyl/viridifloryl dihydroxy platynecine	C15 H27 N O7	-1.64	333.1782	4.35	3	8	6	82.4	65	Single match found
39	PE	trihydroxytrachelanthamidine (or isomer)	trachelantyl/viridifloryl dihydroxy platynecine	C15 H27 N O7	4.12	333.1801	4.37	3	10	0			Single match found
40	PE	trihydroxytrachelanthamidine (or isomer)	trachelantyl/viridifloryl dihydroxy platynecine	C15 H27 N O7	-1.68	333.1782	4.59	3	8	3	81.1	50.7	Single match found
41	PE	tetrahydroxytrachelanthamidine (or isomer)	trachelantyl/viridifloryl trihydroxy platynecine	C15 H27 N O8	-0.73	349.1734	3.10	3	1	4	76.4	48	Single match found
42	PE	tetrahydroxytrachelanthamidine (or isomer)	trachelantyl/viridifloryl trihydroxy platynecine	C15 H27 N O8	-0.92	349.1734	3.60	3	1	4	83.3	52	Single match found
43	PE	tetrahydroxytrachelanthamidine (or isomer)	trachelantyl/viridifloryl trihydroxy platynecine	C15 H27 N O8	-0.82	349.1734	3.72	3	1	3	79.9	50	Single match found
44	ME	heleurine	heliotridyl/curassavoyl supinidine-type retronecine/heliotridine	C16 H27 N O4	-1.29	297.1936	9.62	2	32	0			Single match found
45	ME	heleurine N-oxide	heliotridyl/curassavoyl retronecine/heliotridine	C16 H27 N O5	-3.01	313.1880	6.97	2	22	0			Single match found
46	ME	heliotrine (isomer)	heliotridyl/curassavoyl retronecine/heliotridine	C16 H27 N O5	-1.97	313.1883	7.91	3	22	0			Single match found
47	ME	heliotrine (isomer)	heliotridyl/curassavoyl retronecine/heliotridine	C16 H27 N O5	-3.22	313.1879	8.15	3	22	0			Single match found
48	ME	heliotrine (isomer)	heliotridyl/curassavoyl retronecine/heliotridine	C16 H27 N O5	-3.22	313.1879	8.30	3	22	0			Single match found
49	ME	heliotrine	heliotridyl/curassavoyl retronecine/heliotridine	C16 H27 N O5	-2.77	313.1881	8.61	1	22	0			Single match found
50	ME	heliotrine N-oxide (isomer)	heliotridyl/curassavoyl N-oxide/hydroxy retronecine/heliotridine	C16 H27 N O6	-2.34	329.1831	5.04	3	7	0			Single match found
51	ME	heliotrine N-oxide (isomer)	heliotridyl/curassavoyl N-oxide/hydroxy retronecine/heliotridine	C16 H27 N O6	-1.32	329.1834	5.40	3	7	2	63.4	43.1	Single match found
52	ME	heliotrine N-oxide (isomer)	heliotridyl/curassavoyl N-oxide/hydroxy retronecine/heliotridine	C16 H27 N O6	-2.41	329.1830	5.70	3	7	2	65.9	47.3	Single match found
53	ME	heliotrine N-oxide	heliotridyl/curassavoyl N-oxide/hydroxy retronecine/heliotridine	C16 H27 N O6	-2.80	329.1829	5.95	1	7	0			Single match found
54	ME	europine (isomer)	heliotridyl/curassavoyl N-oxide/hydroxy retronecine/heliotridine	C16 H27 N O6	1.35	329.1843	6.40	3	7	0			Single match found
55	ME	europine	heliotridyl/curassavoyl N-oxide/hydroxy retronecine/heliotridine	C16 H27 N O6	-2.66	329.1830	6.58	1	7	0			Single match found
56	ME	europine (isomer)	heliotridyl/curassavoyl N-oxide/hydroxy retronecine/heliotridine	C16 H27 N O6	0.09	329.1839	6.81	3	7	0			Single match found

57	ME	europine (isomer)	heliotridyl/curassavoyl N-oxide/hydroxy retronecine/heliotridine	C16 H27 N O6	-2.17	329.1831	7.54	3	7	0			Single match found
58	ME	europine (isomer)	heliotridyl/curassavoyl N-oxide/hydroxy retronecine/heliotridine	C16 H27 N O6	-2.17	329.1831	7.82	3	7	0			Single match found
59	ME	europine (isomer)	heliotridyl/curassavoyl N-oxide/hydroxy retronecine/heliotridine	C16 H27 N O6	-2.11	329.1831	7.91	3	7	0			Single match found
60	ME	europine (isomer)	heliotridyl/curassavoyl N-oxide/hydroxy retronecine/heliotridine	C16 H27 N O6	0.42	329.1840	8.39	3	7	0			Single match found
61	ME	europine N-oxide (isomer)	heliotridyl/curassavoyl dihydroxy retronecine/heliotridine	C16 H27 N O7	0.02	345.1788	3.48	3	6	0			Single match found
62	ME	europine N-oxide (isomer)	heliotridyl/curassavoyl dihydroxy retronecine/heliotridine	C16 H27 N O7	-2.34	345.1779	3.76	3	6	0			Single match found
63	ME	europine N-oxide	heliotridyl/curassavoyl dihydroxy retronecine/heliotridine	C16 H27 N O7	-2.45	345.1779	4.03	1	6	0			Single match found
64	ME	hydroxyeuropine (or isomer)	heliotridyl/curassavoyl dihydroxy retronecine/heliotridine	C16 H27 N O7	-1.93	345.1781	5.53	3	6	1	60.4	46.3	Single match found
65	ME	hydroxyeuropine (or isomer)	heliotridyl/curassavoyl dihydroxy retronecine/heliotridine	C16 H27 N O7	-1.48	345.1782	5.62	3	6	0			Single match found
66	ME	hydroxyeuropine (or isomer)	heliotridyl/curassavoyl dihydroxy retronecine/heliotridine	C16 H27 N O7	-1.67	345.1782	5.85	3	6	0			Single match found
67	PE	dihydroheliotrine (or isomer)	heliotridyl/curassavoyl platynecine	C16 H29 N O5	-1.74	315.2040	7.18	3	29	1	76.2	56.6	Single match found
68	PE	dihydroheliotrine (or isomer)	heliotridyl/curassavoyl platynecine	C16 H29 N O5	-0.27	315.2045	7.42	3	29	2	78.3	59.5	Single match found
69	PE	dihydroheliotrine (or isomer)	heliotridyl/curassavoyl platynecine	C16 H29 N O5	-1.07	315.2042	7.81	3	29	2	73	52.2	Single match found
70	PE	dihydroheliotrine N-oxide (or isomer)	heliotridyl/curassavoyl N-oxide/hydroxy (lasiocarpine-type) platynecine	C16 H29 N O6	-0.70	331.1993	5.48	3	9	1	68.3	8.4	Single match found
71	PE	dihydroheliotrine N-oxide (or isomer)	heliotridyl/curassavoyl N-oxide/hydroxy (lasiocarpine-type) platynecine	C16 H29 N O6	-1.39	331.1990	5.85	3	9	2	70.6	8.5	Single match found
72	PE	dihydroeuropine (or isomer)	heliotridyl/curassavoyl N-oxide/hydroxy (lasiocarpine-type) platynecine	C16 H29 N O6	-2.70	331.1986	6.04	3	9	1	60.4	8	Single match found
73	PE	dihydroeuropine (or isomer)	heliotridyl/curassavoyl N-oxide/hydroxy (lasiocarpine-type) platynecine	C16 H29 N O6	-2.51	331.1987	6.44	3	9	1	63	8.1	Single match found
74	PE	dihydroeuropine (or isomer)	heliotridyl/curassavoyl N-oxide/hydroxy (lasiocarpine-type) platynecine	C16 H29 N O6	-1.13	331.1991	7.25	3	9	1	65	8.2	Single match found
75	PE	dihydroeuropine (or isomer)	heliotridyl/curassavoyl N-oxide/hydroxy (lasiocarpine-type) platynecine	C16 H29 N O6	-0.95	331.1992	7.43	3	9	2	65.8	8.3	Single match found
76	PE	dihydroeuropine N-oxide (or isomer)	heliotridyl/curassavoyl dihydroxy platynecine	C16 H29 N O7	-0.11	347.1944	4.05	3	1	0			Single match found
77	PE	hydroxydihydroeuropine (or isomer)	heliotridyl/curassavoyl dihydroxy platynecine	C16 H29 N O7	-0.03	347.1944	5.58	3	1	1	69.3	8.5	Single match found
78	PE	hydroxydihydroeuropine (or isomer)	heliotridyl/curassavoyl dihydroxy platynecine	C16 H29 N O7	-0.87	347.1941	6.21	3	1	4	77.2	8.9	Single match found
79	PE	hydroxydihydroeuropine (or isomer)	heliotridyl/curassavoyl dihydroxy platynecine	C16 H29 N O7	-0.84	347.1941	6.38	3	1	3	77.2	8.9	Single match found
80	PE	hydroxydihydroeuropine (or isomer)	heliotridyl/curassavoyl dihydroxy platynecine	C16 H29 N O7	0.30	347.1945	6.46	3	1	0			Single match found
81	PE	hydroxydihydroeuropine N-oxide (or isomer)	heliotridyl/curassavoyl trihydroxy platynecine	C16 H29 N O8	-1.25	363.1889	4.55	3	11	3	62.8	43	Single match found
82	PE	hydroxydihydroeuropine N-oxide (or isomer)	heliotridyl/curassavoyl trihydroxy platynecine	C16 H29 N O8	3.45	363.1906	4.74	3	12	0			Single match found
83	PE	hydroxydihydroeuropine N-oxide (or isomer)	heliotridyl/curassavoyl trihydroxy platynecine	C16 H29 N O8	3.95	363.1908	5.07	3	12	0			Single match found
84	PE	acetyltrachelanthamidine (or isomer)	acetyl+trachelantyl/virifloryl supinidine-type platynecine	C17 H29 N O5	-0.60	327.2044	6.42	3	2	1	60.8	8	Single match found
85	PE	acetyltrachelanthamidine (or isomer)	acetyl+trachelantyl/virifloryl supinidine-type platynecine	C17 H29 N O5	-0.21	327.2045	10.08	3	2	0			Single match found
86	PE	acetyltrachelanthamidine N-oxide (or isomer)	acetyl+trachelantyl/virifloryl platynecine	C17 H29 N O6	-1.22	343.1991	4.43	3	2	0			Single match found
87	PE	hydroxyacetyltrachelanthamidine (or isomer)	acetyl+trachelantyl/virifloryl platynecine	C17 H29 N O6	0.13	343.1995	7.35	3	2	0			Single match found
88	DE	heliotridine 1,7-diester	diangeloyl/tigloyl/senecioyl N-oxide/hydroxy retronecine/heliotridine	C18 H25 N O5	-3.03	335.1723	11.45	3	41	0			Single match found
89	DE	heliotridine 1,7-diester	diangeloyl/tigloyl/senecioyl N-oxide/hydroxy retronecine/heliotridine	C18 H25 N O5	-2.20	335.1725	11.52	3	41	0			Single match found
90	DE	heliotridine 1,7-diester	diangeloyl/tigloyl/senecioyl N-oxide/hydroxy retronecine/heliotridine	C18 H25 N O5	-2.60	335.1724	12.12	3	41	0			Single match found
91	DE	heliotridine 1,7-diester N-oxide	diangeloyl/tigloyl/senecioyl dihydroxy retronecine/heliotridine	C18 H25 N O6	-0.74	351.1679	10.50	3	35	1	69.1	8.5	Single match found
92	DE	heliotridine 1,7-diester	diangeloyl/tigloyl/senecioyl dihydroxy platynecine	C18 H27 N O6	0.01	353.1838	11.13	3	17	4	81.2	50.8	Multiple matches found
93	ME	5'-acetyleuropine	acetyl+heliotridyl/curassavoyl+N-oxide/hydroxy retronecine/heliotridine	C18 H29 N O7	-1.69	371.1938	8.59	2	3	0			Multiple matches found
94	DE	7-acetyleuropine (or isomer)	acetyl+heliotridyl/curassavoyl+N-oxide/hydroxy retronecine/heliotridine	C18 H29 N O7	-1.85	371.1937	8.86	2	3	0			Multiple matches found
95	ME	5'-acetyleuropine N-oxide (isomer)	acetyl+heliotridyl/curassavoyl+dihydroxy retronecine/heliotridine	C18 H29 N O8	-0.62	387.1891	5.58	3	28	2	74.4	54.1	Single match found
96	ME	5'-acetyleuropine N-oxide	acetyl+heliotridyl/curassavoyl+dihydroxy retronecine/heliotridine	C18 H29 N O8	-1.36	387.1888	6.34	2	28	0			Single match found
97	DE	7-acetyleuropine N-oxide (or isomer)	acetyl+heliotridyl/curassavoyl+dihydroxy retronecine/heliotridine	C18 H29 N O8	-2.28	387.1884	6.52	2	12	0			Single match found
98	PE	acetyldihydroheliotrine N-oxide (or isomer)	angeloyl/tigloyl/senecioyl+trachelantyl/viridifloryl N-oxide/hydroxy retronecine/heliotridine	C18 H31 N O7	-0.02	373.2101	5.35	3	5	0			Single match found
99	DE	heliosupine (isomer)	angeloyl/tigloyl/senecioyl+trachelantyl/viridifloryl N-oxide/hydroxy retronecine/heliotridine	C20 H31 N O7	-0.19	397.2100	10.71	3	7	0			Multiple matches found
100	DE	heliosupine	angeloyl/tigloyl/senecioyl+trachelantyl/viridifloryl N-oxide/hydroxy retronecine/heliotridine	C20 H31 N O7	-0.10	397.2100	10.79	1	7	0			Multiple matches found
101	DE	heliosupine N-oxide (isomer)	angeloyl/tigloyl/senecioyl+trachelantyl/viridifloryl dihydroxy/N-oxide retronecine/heliotridine	C20 H31 N O8	-0.62	413.2047	7.39	3	4	4	83.2	9.2	Multiple matches found
102	DE	heliosupine N-oxide	angeloyl/tigloyl/senecioyl+trachelantyl/viridifloryl dihydroxy/N-oxide retronecine/heliotridine	C20 H31 N O8	-0.65	413.2047	7.55	1	4	2	78.1	8.9	Multiple matches found
103	DE	echimidine N-oxide	angeloyl/tigloyl/senecioyl+trachelantyl/viridifloryl dihydroxy/N-oxide retronecine/heliotridine	C20 H31 N O8	-0.60	413.2047	7.86	1	4	6	79.7	9	Multiple matches found
104	DE	7-angeloylheliotrine	angeloyl/tigloyl/senecioyl+heliotridyl/curassavoyl retronecine/heliotridine	C21 H33 N O6	0.81	395.2311	12.38	2	8	0			Single match found
105	DE	7-angeloylheliotrine N-oxide	angeloyl/tigloyl/senecioyl+heliotridyl/curassavoyl N-oxide/hydroxy retronecine/heliotridine	C21 H33 N O7	-1.07	411.2253	10.57	2	2	0			Single match found
106	DE	7-tigloyleuropine	angeloyl/tigloyl/senecioyl+heliotridyl/curassavoyl N-oxide/hydroxy retronecine/heliotridine	C21 H33 N O7	-3.17	411.2244	11.45	2	2	0			Single match found
107	DE	lasiocarpine	angeloyl/tigloyl/senecioyl+heliotridyl/curassavoyl N-oxide/hydroxy retronecine/heliotridine	C21 H33 N O7	-3.17	411.2244	11.54	1	2	0			Single match found
108	DE	7-tigloyleuropine N-oxide	angeloyl/tigloyl/senecioyl+heliotridyl/curassavoyl dihydroxy retronecine/heliotridine	C21 H33 N O8	-1.30	427.2201	8.58	2	1	2	76.7	8.8	Single match found
109	DE	lasiocarpine N-oxide	angeloyl/tigloyl/senecioyl+heliotridyl/curassavoyl dihydroxy retronecine/heliotridine	C21 H33 N O8	-1.28	427.2201	8.77	1	1	2	77.7	8.9	Single match found
110	DE	heliotridine 1,7-diester	angeloyl/tigloyl/senecioyl+heliotridyl/curassavoyl trihydroxy platynecine	C21 H35 N O9	0.63	445.2315	10.65	3	3	0			Multiple matches found
111	DE	methoxylasiocarpine	acetyl+angeloyl/tigloyl/senecioyl+trachelantyl/viridifloryl platynecine	C22 H35 N O7	0.14	425.2414	11.91	3	3	0			Multiple matches found
112	ME	heliotrine glycoside (or isomer)	ditrachelantyl/viridifloryl dihydroxy retronecine/heliotridine	C22 H37 N O10	-0.34	475.2416	7.57	3	2	0			Multiple matches found
113	ME	europine glycoside (or isomer)	ditrachelantyl/viridifloryl trihydroxy retronecine/heliotridine	C22 H37 N O11	0.29	491.2368	5.10	3	4	0			Single match found
114	ME	europine glycoside (or isomer)	ditrachelantyl/viridifloryl trihydroxy retronecine/heliotridine	C22 H37 N O11	0.24	491.2368	5.20	3	4	0			Single match found
115	ME	europine glycoside (or isomer)	ditrachelantyl/viridifloryl trihydroxy retronecine/heliotridine	C22 H37 N O11	-0.95	491.2362	6.03	3	9	0			Single match found
116	DE	5'-acetyl-7-tigloyleuropine	acetyl+angeloyl/tigloyl/senecioyl+heliotridyl/curassavoyl N-oxide/hydroxy retronecine/heliotrid	C23 H35 N O8	1.38	453.2369	11.86	2	5	0			Single match found
117	DE	5'-acetyllasiocarpine	acetyl+angeloyl/tigloyl/senecioyl+heliotridyl/curassavoyl N-oxide/hydroxy retronecine/heliotrid	C23 H35 N O8	-2.54	453.2351	12.12	2	2	0			Single match found
118	DE	5'-acetyl-7-tigloyleuropine N-oxide	acetyl+angeloyl/tigloyl/senecioyl+heliotridyl/curassavoyl dihydroxy retronecine/heliotridine	C23 H35 N O9	-1.31	469.2306	10.33	2	1	0			Multiple matches found
119	DE	5'-acetyllasiocarpine N-oxide	acetyl+angeloyl/tigloyl/senecioyl+heliotridyl/curassavoyl dihydroxy retronecine/heliotridine	C23 H35 N O9	-0.84	469.2308	10.48	2	1	0			Multiple matches found
120	DE	5'-acetyllasiocarpine N-oxide isomer	acetyl+angeloyl/tigloyl/senecioyl+heliotridyl/curassavoyl dihydroxy retronecine/heliotridine	C23 H35 N O9	0.47	469.2314	10.71	3	1	0			Multiple matches found

120 compounds
48 platynecine mono en diesters (PE)
47 heliotridine monoesters (ME)
25 heliotridine diesters (DE)

77 tertiary amines
43 N-oxides

min intensity: 5.00E+05

sample area must be 10x blank area

No	(tentative) name	MS2	max area std	sum area fractions	H europaeum fraction 1 Area: Qex_210924_PAs_102.raw (F102)	H europaeum fraction 2 Area: Qex_210924_PAs_103.raw (F103)	H europaeum fraction 3 Area: Qex_210924_PAs_104.raw (F104)	H europaeum fraction 4 Area: Qex_210924_PAs_105.raw (F105)	H europaeum fraction 5 Area: Qex_210924_PAs_106.raw (F106)	H europaeum fraction 6 Area: Qex_210924_PAs_107.raw (F107)	H europaeum fraction 7 Area: Qex_210924_PAs_108.raw (F108)	H europaeum fraction 8 Area: Qex_210924_PAs_109.raw (F109)	H europaeum fraction 9 Area: Qex_210924_PAs_110.raw (F110)	H europaeum fraction 10 Area: Qex_210924_PAs_111.raw (F111)	WS 1000 ng/ml Area: Qex_210924_PAs_067.raw (F67)	WS 1000 ng/ml Area: Qex_210924_PAs_068.raw (F68)	WS 1000 ng/ml Area: Qex_210924_PAs_113.raw (F113)	WS 1000 ng/ml Area: Qex_210924_PAs_114.raw (F114)
1	angeloyl heliotridine (or isomer)	DDA for preferred ion	5625933	64832583							38598025	6552157	11771660	7910742	661051	709577	5050232	5625933
2	angeloyl heliotridine (or isomer)	DDA for preferred ion	3975147	9662790							7191703	1037918	923302	509868	3975147	3844515		3614163
3	angeloyl heliotridine (or isomer)	DDA for preferred ion	4735708	7369164									6774287	594877	3874361	3747781	3928706	4735708
4	angeloyl heliotridine N-oxide (or isomer)	DDA for preferred ion	3669844	20675168				12749731		1583153	4456934	1885349					3669844	2909766
5	angeloyl heliotridine N-oxide (or isomer)	DDA for preferred ion	32092868	277441928		652173		594551	266625916	971037	6217198	555283	1825768			32092868	29601860	30211592
6	angeloyl heliotridine N-oxide (or isomer)	DDA for preferred ion	1325802	23036220					23036220						1025089		874262	1325802
7	hydroxyangeloyl heliotridine (or isomer)	DDA for preferred ion	1629806	121602552							115837674	746231		5018647	1422643	1406236	1629806	1227956
8	hydroxyangeloyl heliotridine N-oxide (or isome	DDA for preferred ion	2323421	21024887			20326605		698282						2323421	1136313	2169620	2081719
9	dihydroxyangeloyl heliotridine (or isomer)	DDA for preferred ion	0	20023593					20023593									
10	hydroxyangeloyl platynecine N-oxide (or isome	DDA for preferred ion	1020309	13754477					12956427		798050						902266	1020309
11	dihydroxyangeloyl platynecine (or isomer)	DDA for preferred ion	2366293	19339040					19339040						2366293	2105913	1602160	1597368
12	dihydroxyangeloyl platynecine N-oxide (or isor	DDA for preferred ion	549202	13947413			13245567		701846						549202			
13	supinine	DDA for preferred ion	580190	87241979						86311163	930817							580190
14	supinine N-oxide	DDA for preferred ion	925019	67377953				63972810	1814521	1590622							582984	925019
15	echinatine	DDA for preferred ion	3523335075	105178478					99829295	1611667				3737516	3523335075	3471475882	3419007766	3408725911
16	rinderine	DDA for preferred ion	4152293418	905402087			755354	675990	255401159	639481137	6340327	743449		2004671	74314898	4152293418	3819980118	3834097971
17	echinatine N-oxide	DDA for preferred ion	2774504198	86235027			81625307	611766	2008096					1989858	2631520695	2774504198	2507144752	2756328212
18	rinderine N-oxide	DDA for preferred ion	3068179471	820234648		1522029	791457775	8088392	6962588	8403714	2813339			986812	43655750	26635848	3068179471	49364296
19	5'-hydroxyrinderine	DDA for preferred ion	25421024	538709260		1737239		520330295	9067676	518962		6217597		837491	23573542	22110837	23960647	25421024
20	5'-hydroxyrinderine N-oxide	DDA for preferred ion	5905168	11225840			2310086	7549833		1365920					5905168	649171	553437	817008
21	trachelanthamine	DDA for preferred ion	4851610453	3249815					3249815						4739611989	4851610453	9348170	7657685
22	hydroxytrachelanthamidine (or isomer)	DDA for preferred ion	5291368	83603362				81386890	1397478					818994	578798	602159	2243662	5291368
23	hydroxytrachelanthamidine (or isomer)	DDA for preferred ion	1714483	6822501				5360102						1462399	852033	601250	1714483	1164131
24	trachelanthamine N-oxide	DDA for preferred ion	4334478913	3053724				2297203	756522						4334478913	4330349471	4116707743	4027152590
25	hydroxytrachelanthamidine (or isomer)	DDA for preferred ion	15841839	20717218					20717218						8848595	7534335	15841839	14462590
26	hydroxytrachelanthamidine (or isomer)	DDA for preferred ion	2453413	286107369					285468885	638484					943842	1124710	1813618	2453413
27	hydroxytrachelanthamidine (or isomer)	DDA for preferred ion	1018903	13506580					13506580						897068	1003661	825326	1018903
28	hydroxytrachelanthamidine (or isomer)	DDA for preferred ion	2009687	216364298					215516142	848157					999054	1040014	1692970	2009687
29	hydroxytrachelanthamidine (or isomer)	DDA for preferred ion	963905	46826960					46826960						866831	834101	963905	915428
30	hydroxytrachelanthamidine (or isomer)	DDA for preferred ion	1419240	14928319					14928319						520370	532634	1419240	1063938
31	hydroxytrachelanthamidine N-oxide (or isome	DDA for preferred ion	614340	134882916			126840265	1236644	6806007								534594	614340
32	dihydroxytrachelanthamidine (or isomer)	DDA for preferred ion	2794879	83849485			32823217	46426482	4599786								2794879	2632500
33	dihydroxytrachelanthamidine (or isomer)	DDA for preferred ion	648374	66138432			1708512	62079744	2350176								648374	
34	dihydroxytrachelanthamidine (or isomer)	DDA for preferred ion	0	8727634				7854107	873527									
35	dihydroxytrachelanthamidine (or isomer)	DDA for preferred ion	0	169921464				168711846	1209618									
36	dihydroxytrachelanthamidine (or isomer)	DDA for preferred ion	777867	51045435				12234627	38810808						777867	622505	702631	767773
37	trihydroxytrachelanthamidine (or isomer)	DDA for preferred ion	640820	17817708			14146610	3671098							640820	561395		
38	trihydroxytrachelanthamidine (or isomer)	DDA for preferred ion	0	145941288			44316926	100727155	897207									
39	trihydroxytrachelanthamidine (or isomer)	No MS2	0	18785809			16111137	1777465	897207									
40	trihydroxytrachelanthamidine (or isomer)	DDA for preferred ion	0	67667833			2595535	62439161	2633137									
41	tetrahydroxytrachelanthamidine (or isomer)	DDA for preferred ion	0	24407846			24407846											
42	tetrahydroxytrachelanthamidine (or isomer)	DDA for preferred ion	1384613	14654047			10615577	4038470							625534	582264	1112175	1384613
43	tetrahydroxytrachelanthamidine (or isomer)	DDA for preferred ion	653136	9227104			6793988	2433116							653136	622367		639009
44	heleurine	DDA for preferred ion	3947495	726755492					597291			726158201			3909892	3947495	1000289	1296473
45	heleurine N-oxide	DDA for preferred ion	697726	227446853		599893		1913481	166739350	34607385		23586745			527655	517057	547966	697726
46	heliotrine (isomer)	DDA for preferred ion	503900	56378162					592018	39241586	16544558							503900
47	heliotrine (isomer)	DDA for preferred ion	589624	29680641					553481		28592372	534788			589624	557131	522047	582940
48	heliotrine (isomer)	DDA for preferred ion	513624	14391102							13763796	627305						513624
49	heliotrine	DDA for preferred ion	4655003911	31910757893		3104523	3115072	14453475	70537829	12366766	31702734738	73520330	6051009	24874152	4655003911	4456826112	4540858256	4252707283
50	heliotrine N-oxide (isomer)	DDA for preferred ion	0	23421908				22436146	985762									
51	heliotrine N-oxide (isomer)	DDA for preferred ion	0	25795044			1017002	12585273	12192769									
52	heliotrine N-oxide (isomer)	DDA for preferred ion	0	26702448			1024325	15094636	9953986		629500							
53	heliotrine N-oxide	DDA for preferred ion	4021657943	19148631654		53779163	7567154	70757487	18170847849	12212318	806740983	14993434	1993129	9740138	3924738304	4021657943	3730951455	3655493343
54	europine (isomer)	No MS2	3414267	210760190			547265		208510425	501644	1200855				2104824	2158098	2992957	3414267
55	europine	DDA for preferred ion	3821140024	29170497244	951777	76023790	52537143	65095594	27858087878	118510390	702064857	60807056	190094832	46323926	3667776822	3821140024	3284525784	3413105011
56	europine (isomer)	No MS2	44035666	707634368		559666	2378130	2205112	658296464	12358299	6056100	678577	19351940	5750081	7955574	13143212	44035666	31077717

57	europine (isomer)	DDA for preferred ion	946033	28208567				15643979	12042093	522496				892395	838239	946033	791474
58	europine (isomer)	DDA for preferred ion	964303	22667306				14630214	6887967	1149124				783653	895004	964303	832412
59	europine (isomer)	DDA for preferred ion	907397	182734661	1034125	2998609	7685134	20453565	112875144	19930950	14363458	887005	2506671	759806	760217	747530	907397
60	europine (isomer)	No MS2	593940	34008842				33478596	530246					562842	593940	579089	543562
61	europine N-oxide (isomer)	No MS2	0	59310027		59310027											
62	europine N-oxide (isomer)	DDA for preferred ion	3174315996	131795171		131151177		643994						3174315996	511407	601770	794125
63	europine N-oxide	DDA for preferred ion	3036207244	25817933662	152442068	24514896749	105128102	959756850	13654128	52745494	7397424	4012309	7900538	33730718	20964585	3036207244	2874898862
64	hydroxyeuropine (or isomer)	DDA for preferred ion	566906	23652022		3124185		20527837								566906	521139
65	hydroxyeuropine (or isomer)	DDA for preferred ion	0	9668534		1551026	6842256	1275252									
66	hydroxyeuropine (or isomer)	DDA for preferred ion	652815	181460091	3558948	8492719	44545022	80436380	21981445	11330019	9294175	733281	1088101	624516	652815		
67	dihydroheliotrine (or isomer)	DDA for preferred ion	734860	43073820					43073820					734860			
68	dihydroheliotrine (or isomer)	DDA for preferred ion	0	21658351					21658351								
69	dihydroheliotrine (or isomer)	DDA for preferred ion	551254	409967893				532024	340934442	67627513	873914					525578	551254
70	dihydroheliotrine N-oxide (or isomer)	DDA for preferred ion	571248	13232512			12222497		1010015					547635	571248		536259
71	dihydroheliotrine N-oxide (or isomer)	DDA for preferred ion	751632	69788043				69788043						719050	751632		
72	dihydroeuropine (or isomer)	DDA for preferred ion	0	123436789			729276	110659384	9555297	2492831							
73	dihydroeuropine (or isomer)	DDA for preferred ion	0	7762710				7762710									
74	dihydroeuropine (or isomer)	DDA for preferred ion	0	4759365					4759365								
75	dihydroeuropine (or isomer)	DDA for preferred ion	0	45897162					45367918		529244						
76	dihydroeuropine N-oxide (or isomer)	DDA for preferred ion	0	5896654		5071302		825351									
77	hydroxydihydroeuropine (or isomer)	DDA for preferred ion	0	18036585			10888076	7148509									
78	hydroxydihydroeuropine (or isomer)	DDA for preferred ion	1054540	77858715				76700220		1158495				1054540	926039	644143	874961
79	hydroxydihydroeuropine (or isomer)	DDA for preferred ion	0	99033650				99033650									
80	hydroxydihydroeuropine (or isomer)	No MS2	0	29277326				27926094	787032	564200							
81	hydroxydihydroeuropine N-oxide (or isomer)	DDA for preferred ion	0	94987190		20869210	72648013	1469967									
82	hydroxydihydroeuropine N-oxide (or isomer)	No MS2	0	225374623		930790	221921741	2522093									
83	hydroxydihydroeuropine N-oxide (or isomer)	No MS2	0	11719173			10733693	985480									
84	acetyltrachelanthamidine (or isomer)	DDA for preferred ion	3941314	20554639				19346787		1207852				3814430	3941314	3768538	3653908
85	acetyltrachelanthamidine (or isomer)	DDA for preferred ion	0	30502951							30502951						
86	acetyltrachelanthamidine N-oxide (or isomer)	DDA for preferred ion	0	54132702		1022205	49557826	3552670									
87	hydroxyacetyltrachelanthamidine (or isomer)	DDA for preferred ion	0	7581128					6988214		592915						
88	heliotridine 1,7-diester	DDA for preferred ion	6899763	4644156								4644156		3790442	3822143	6899763	4496561
89	heliotridine 1,7-diester	DDA for preferred ion	101305548	69369155								63145248	6223908	52671827	34524037	101305548	43828188
90	heliotridine 1,7-diester	DDA for preferred ion	1979678	297371027									297371027	1893404	1708309	1503977	1979678
91	heliotridine 1,7-diester N-oxide	DDA for preferred ion	3100381	8716539							7810240		906299	3100381	2449954	2378746	2207263
92	heliotridine 1,7-diester	DDA for preferred ion	3571187	11194087							948330	5131489	5114268	512824	551299	3571187	2906742
93	5'-acetyleuropine	DDA for preferred ion	6970414	548827718				1086943	846298	520778848	2686639	681155	22747835	6288402	6970414	6765858	6261633
94	7-acetyleuropine (or isomer)	DDA for preferred ion	1968805	303774976						299294803	553248		3926925	1968805	1913334		877339
95	5'-acetyleuropine N-oxide (isomer)	DDA for preferred ion	0	55187561			2481780	51077556		1628225							
96	5'-acetyleuropine N-oxide	DDA for preferred ion	5184808	601393845	1049802		2854681	575701580	1014805	16075237	3764155		933586		4587173	5136041	5184808
97	7-acetyleuropine N-oxide (or isomer)	No MS2	0	7815893				7815893									
98	acetyldihydroheliotrine N-oxide (or isomer)	DDA for preferred ion	0	7871933			7871933										
99	heliosupine (isomer)	DDA for preferred ion	49802465	20867280							20867280			49802465	39478460	40609002	39938064
100	heliosupine	DDA for preferred ion	5061937186	275184234					754187		272116296	1409678	904073	5061937186	4865079968	4884479206	4692954129
101	heliosupine N-oxide (isomer)	DDA for preferred ion	19984267	6989272					6989272					14011573	19984267	16301243	16385201
102	heliosupine N-oxide	DDA for preferred ion	4212389844	93290451			2188109	3919428	83305069		3365312		512533	4212389844	4075101265	4184961442	4137231056
103	echimidine N-oxide	DDA for preferred ion	3908596122	5252555					5252555					3774327452	3908596122	3865427923	3905044520
104	7-angeloylheliotrine	DDA for preferred ion	0	111568297									111568297				
105	7-angeloylheliotrine N-oxide	DDA for preferred ion	0	15255583							13940506		1315077				
106	7-tigloyleuropine	DDA for preferred ion	73514404	987802668				1665825	568014	812669		907769122	76987038	28807464	26867620	73514404	23688729
107	lasiocarpine	No MS2	3252233522	8390594207	530756		2391393	14137669	4820672	6897028	4302017	7704134345	653380327	1274425068	1188607516	3252233522	1047975287
108	7-tigloyleuropine N-oxide	DDA for preferred ion	99111471	182074178			752552	693193		161696555	1675675	14525536	2730666	95265120	98195966	99111471	97371308
109	lasiocarpine N-oxide	DDA for preferred ion	4706174248	3080314724	4021978	997532	23283552	37002788		2761986584	25119625	192045355	35857311	4706174248	4516605022	4526804621	4154671918
110	heliotridine 1,7-diester	DDA for preferred ion	1453478	16093411							16093411			1453478	1172111		643538
111	methoxylasiocarpine	DDA for preferred ion	568532	6890726									6890726	568532	538237	518351	512201
112	heliotrine glycoside (or isomer)	DDA for preferred ion	637703	11587554					11587554							637703	593426
113	europine glycoside (or isomer)	DDA for preferred ion	0	28759063			28759063										
114	europine glycoside (or isomer)	DDA for preferred ion	0	6787905			6787905										
115	europine glycoside (or isomer)	DDA for preferred ion	0	10071289				10071289									
116	5'-acetyl-7-tigloyleuropine	No MS2	0	130713020									130713020				
117	5'-acetyllasiocarpine	DDA for preferred ion	2405203	1898212586				519145		883225	2995476		1893814741	2405203	2040668	2311820	2399492
118	5'-acetyl-7-tigloyleuropine N-oxide	DDA for preferred ion	0	24064154							21395317		2668837				
119	5'-acetyllasiocarpine N-oxide	DDA for preferred ion	0	372923839			1996142	4070840			326137588		40719269				
120	5'-acetyllasiocarpine N-oxide isomer	No MS2	1559099	31106394							27589920		3516474	1186972	1395621	1559099	1406419

	Total counts	H europaeum fraction 1	H europaeum fraction 2	H europaeum fraction 3	H europaeum fraction 4	H europaeum fraction 5	H europaeum fraction 6	H europaeum fraction 7	H europaeum fraction 8	H europaeum fraction 9	H europaeum fraction 10
120 compounds	132549956375	951777	300616156	26008681927	2004057429	50820666951	1730366304	37390293975	1733559534	9137904605	3422857716
48 platynecine mono en diesters (PE)	2977691992	0	0	321498687	949247167	1122695687	475621093	73848941	32499023	0	2281393
47 heliotridine monoesters (ME)	113220180975	951777	296063422	25686185709	1024198514	49628146485	1153055442	34084874171	956150271	245099676	145455507
25 heliotridine diesters (DE)	16352083409	0	4552734	997532	30611748	69824779	101689769	3231570863	744910240	8892804929	3275120816
36 platynecine mono en diesters tertiary amines	2329050631	0	0	153519348	570757617	1022331281	474611078	73050892	32499023	0	2281393
27 heliotridine monoesters tertiary amines	65741990575	951777	86018293	70824292	690537589	29357298075	1077652361	33193567260	903967881	237268470	123904576
14 heliotridine diesters tertiary amines	12524279829	0	530756	0	2391393	16322638	6142873	307887724	317876057	8686234037	3186894350
12 platynecine mono en diesters N-oxides	648641361	0	0	167979339	378489550	100364407	1010015	798050	0	0	0
20 heliotridine monoesters N-oxides	47478190400	0	210045129	25615361416	333660924	20270848409	75403082	891306911	52182391	7831206	21550932
11 heliotridine diesters N-oxides	3827803580	0	4021978	997532	28220355	53502141	95546896	2923683139	427034183	206570891	88226466

	percentage	H europaeum fraction 1	H europaeum fraction 2	H europaeum fraction 3	H europaeum fraction 4	H europaeum fraction 5	H europaeum fraction 6	H europaeum fraction 7	H europaeum fraction 8	H europaeum fraction 9	H europaeum fraction 10
120 compounds	100.0%	0.0%	0.2%	19.6%	1.5%	38.3%	1.3%	28.2%	1.3%	6.9%	2.6%
48 platynecine mono en diesters (PE)	2.2%	0.0%	0.0%	0.2%	0.7%	0.8%	0.4%	0.1%	0.0%	0.0%	0.0%
47 heliotridine monoesters (ME)	85.4%	0.0%	0.2%	19.4%	0.8%	37.4%	0.9%	25.7%	0.7%	0.2%	0.1%
25 heliotridine diesters (DE)	12.3%	0.0%	0.0%	0.0%	0.0%	0.1%	0.1%	2.4%	0.6%	6.7%	2.5%
36 platynecine mono en diesters tertiary amines	1.8%	0.0%	0.0%	0.1%	0.4%	0.8%	0.4%	0.1%	0.0%	0.0%	0.0%
27 heliotridine monoesters tertiary amines	49.6%	0.0%	0.1%	0.1%	0.5%	22.1%	0.8%	25.0%	0.7%	0.2%	0.1%
14 heliotridine diesters tertiary amines	9.4%	0.0%	0.0%	0.0%	0.0%	0.0%	0.0%	0.2%	0.2%	6.6%	2.4%
12 platynecine mono en diesters N-oxides	0.5%	0.0%	0.0%	0.1%	0.3%	0.1%	0.0%	0.0%	0.0%	0.0%	0.0%
20 heliotridine monoesters N-oxides	35.8%	0.0%	0.2%	19.3%	0.3%	15.3%	0.1%	0.7%	0.0%	0.0%	0.0%
11 heliotridine diesters N-oxides	2.9%	0.0%	0.0%	0.0%	0.0%	0.0%	0.1%	2.2%	0.3%	0.2%	0.1%
